# Atlantic cod (*Gadus morhua*) MHC I localizes to endolysosomal compartments independently of cytosolic sorting signals

**DOI:** 10.3389/fcell.2023.1050323

**Published:** 2023-01-25

**Authors:** Synne Arstad Bjørnestad, Monica Hongrø Solbakken, Kjetill S. Jakobsen, Sissel Jentoft, Oddmund Bakke, Cinzia Progida

**Affiliations:** ^1^ Section of Physiology and Cell Biology, Department of Biosciences, University of Oslo, Oslo, Norway; ^2^ Centre for Ecological and Evolutionary Synthesis (CEES), Department of Biosciences, University of Oslo, Oslo, Norway

**Keywords:** Atlantic cod (Gadus morhua), MHC I, endolysosomes, sorting signals, cross-presentation

## Abstract

Major histocompatibility complex (MHC) class I and II are crucial for the adaptive immune system because they are involved in peptide presentation to T cells. Until recently, it was believed that MHC genes and their associated immune components had been conserved since their evolutionary emergence in jawed fish. However, sequencing of the Atlantic cod (*Gadus morhua*) genome revealed a loss of MHC class II genes, and an extreme expansion of MHC class I genes. These findings lead to the hypothesis that a loss of the MHC class II pathway coincided with a more versatile use of MHC class I, but so far there is no direct experimental evidence in support of this. To gain a deeper understanding of the function of the expanded MHC class I, we selected five MHC class I gene variants representing five of the six clades identified in previous studies and investigated their intracellular localization in human and Atlantic cod larval cells. Intriguingly, we uncovered that all selected MHC class I variants localize to endolysosomal compartments in Atlantic cod cells. Additionally, by introducing point mutations or deletions in the cytosolic tail, we found that hypothetical sorting signals in the MHC class I cytosolic tail do not influence MHC class I trafficking. Moreover, we demonstrated that in Atlantic cod, tapasin and MHC class I colocalize on endolysosomes suggesting that peptide-loading assistance and stabilization of MHC class I occurs outside the endoplasmic reticulum. Altogether, our results demonstrate that MHC class I from Atlantic cod is sorted to the endolysosomal system, which may indicate that it interacts with exogenous peptides for potential cross presentation.

## Introduction

Major histocompatibility complex (MHC) class I and II are crucial components of the adaptive immune system in jawed vertebrates including teleosts (Litman et al., 2010; Flajnik, 2018), and have been acknowledged to be ubiquitous features of the adaptive immune system since they emerged approximately 500 million years ago. However, several reports have uncovered that there are teleost lineages where the entire (or parts of the) MHC II pathway is functionally lost (Star et al., 2011; Haase et al., 2013; Dubin et al., 2019; Roth et al., 2020; Swann et al., 2020). In Atlantic cod (*Gadus morhua*), MHC II-associated gene loss includes the MHC class II interacting molecule CD4, necessary for T cell activation as well as the chaperone invariant chain (Ii), which facilitates MHC II assembly, transport and peptide loading (Star et al., 2011). These findings imply that Atlantic cod do not have a functional MHC II pathway. The lack of this pathway has been firmly established with improved Atlantic cod genome assemblies (Tørresen et al., 2017), genome sequencing of Atlantic haddock (Tørresen et al., 2018), as well as large scale teleost genome assemblies demonstrating that the loss of the MHC II pathway occurred in the range of 80–100 Mya at the very base of the Gadiformes radiation (Tørresen et al., 2018).

In contrast to the loss of MHC II, Atlantic cod is characterized by an expanded MHC I gene repertoire consisting of 80–100 predicted gene copies (Star et al., 2011). To date, little is known on the functional significance of this large MHC I gene repertoire. It is hypothesized that the expansion of Atlantic cod MHC I gene repertoire have given rise to multiple MHC I gene copies that have evolved to function more like MHC class II molecules (Malmstrom et al., 2013; Malmstrom et al., 2016). MHC class I and II present peptides at the cell surface to cytotoxic CD8^+^ and helper CD4^+^ T cells respectively. While class I normally presents endogenously derived peptides, typically of viral or tumoral origin, class II usually presents exogenous peptides derived from infectious organisms such as bacteria and endoparasites (Neefjes et al., 2011). A link between the two pathways exists called cross-presentation, whereby MHC class I presents exogenous antigens to cytotoxic CD8^+^ T cells (Neefjes et al., 2011). MHC II is transported to the endosomal compartments known as the MHC class II compartments (MIIC) by the chaperone Ii; MHC I instead follows the secretory pathway to the plasma membrane (Neefjes et al., 2011). During cross-presentation, MHC I molecules are able to divert from their normal trafficking pathway and can enter the MIIC for binding of exogenous antigens (Neefjes et al., 2011).

So far, most of the inference of gene function for the expanded gene repertoire in Atlantic cod have been conducted *in silico* (including sequence alignments, homology, and gene trees analyses) (Malmstrom et al., 2013; Malmstrom et al., 2016). From one such analyses, Malmstrøm et al., 2013 (Tørresen et al., 2018) identified putative endosomal sorting signals in the cytosolic tail of a subset of MHC I gene copies, and hypothesized that the expanded Atlantic cod MHC I gene repertoire have evolved to allow a more versatile use of MHC I through cross-presentation, thus functioning more like MHC II. These signals consist of a tyrosine-based motif, a motif required for cross-presentation by MHC I molecules (Lizée et al., 2003), and of a dileucine-based signal which is normally associated with classical MHC II function (Pieters et al., 1993; Lizée et al., 2005; Basha et al., 2008). In this study, we aimed to experimentally validate whether the motifs identified in the cytosolic tail of Atlantic cod MHC I function as sorting signals and compare the cellular localization of Atlantic cod MHC I to human MHC I.

To address these questions, we selected five Atlantic cod MHC I gene copies (variant 1–5) and investigated their trafficking in both human and Atlantic cod cell lines by using high resolution microscopy. Our results demonstrate that, differently from mammalian MHC I, Atlantic cod MHC I variants primary localize to the endolysosomal compartments. We furthermore show that this localization is independent of putative cytosolic sorting motifs as neither point mutations nor deletions introduced in these motifs change the intracellular localization of Atlantic cod MHC I. Finally, we show that MHC I and tapasin not only interact, but also co-localize on endolysosomes in Atlantic cod cells. As exogenous antigens are degraded in this pathway, this could facilitate loading of exogenous peptide antigens for cross-presentation.

## Results

### Atlantic cod MHC I variants localize to the endolysosomal system in MelJuSo cells

**FIGURE 1:**
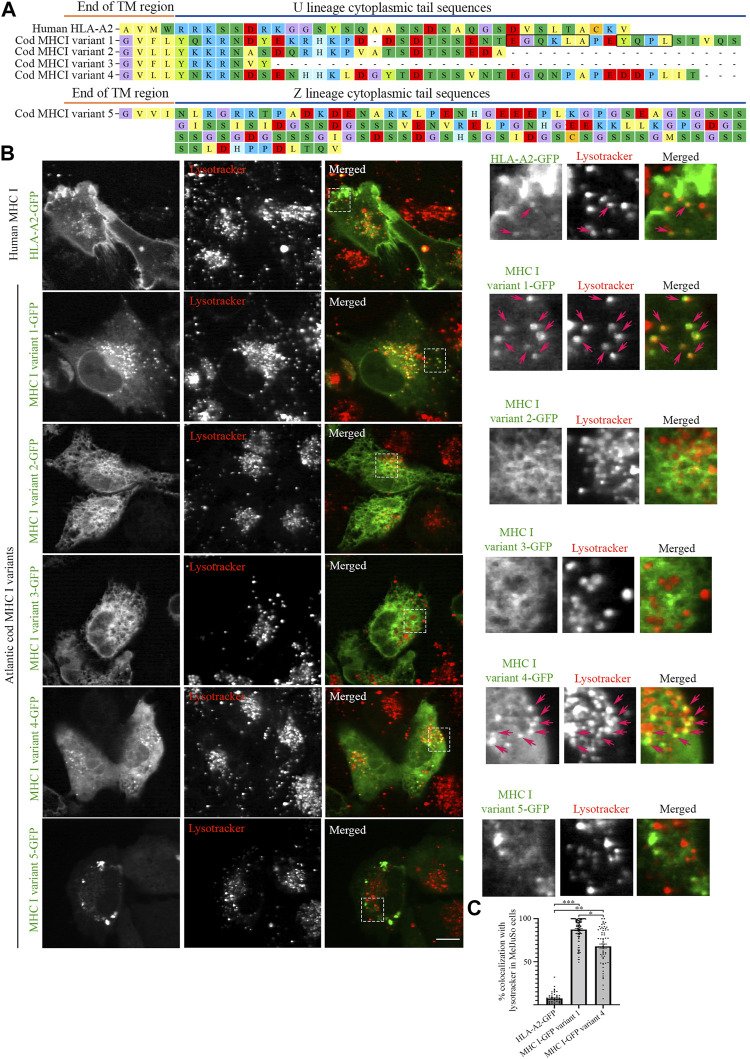
Intracellular localization of human MHC I and five Atlantic cod MHC I variants in MelJuSo cells. **(A)** Amino acid sequences of the cytosolic tails of human HLA-A2 and five selected Atlantic cod MHC I variants (four classical U-lineage MHC I variants and one non-classical Z-lineage MHC I variant). The sequences were manually curated and aligned with MEGA7. Hypothetical dileucine (EGQKLA) and tyrosine (YQPL) sorting signals in MHC I variant 1 are boxed. **(B)** Representative images of MelJuSo cells transiently transfected with human HLA-A2-GFP, or the five selected Atlantic cod MHC I-GFP variants (green). Colocalization between green and red channels results in yellow signal in the merged panels. Cells were stained with lysotracker red and imaged using an Andor Revolution XD Spinning Disk microscope. Scale bar: 10 μm. Magnification of boxed areas are shown to the right of each image. Arrows indicate colocalization with lysotracker. **(C)** The graph represents the percentage of human HLA-A2-GFP, Atlantic cod MHC I-GFP variant 1 or variant 4 vesicles positive for lysotracker red. Colocalization was done by object-based analysis using ImageJ software. Values represent the mean ± s.e.m. from at least three independent experiments. *n* ≥ 52 cells. *p < 0.05, **p < 0.01, ***p < 0.001 (Two-tailed paired Student *t*-test).

To characterize the cellular localization of the MHC I repertoire in Atlantic cod, five Atlantic cod MHC I gene variants were selected based on a phylogenetic tree generated from all cDNA clones from Atlantic cod MHC I U and Z lineage representatives (Malmstrom et al., 2013; Tørresen et al., 2018; Kirubakaran et al., 2020; Matschiner et al., 2022) (https://www.ncbi.nlm.nih.gov/data-hub/genome/GCF_902167405.1/, see *Materials and Methods*, Supplementary Figure S1). Four gene variants were selected from the classical U-lineage and one gene variant was selected from the non-classical Z-lineage (Grimholt et al., 2015) (Figure 1A). The selection was made to best represent the most common tail configurations in term of amino acid sequence and length of the cytosolic tail domain. Variant 1 contains both a putative dileucine- and tyrosine-based sorting signal in its tail. Variant 2 is of intermediate length. Variant 3 is a short tail representative, and variant 4 is a long version that also contains a tyrosine together with an acidic amino acid tract. Finally, an intermediate length non-classical Z-lineage was chosen as variant 5. All selected Atlantic cod MHC I gene variants were C-terminally tagged with GFP, and the classical human MHC I gene variant, the HLA-A2 isoform, was included in the study for comparison. C-terminal GFP tagging was chosen to ensure recognition of the N-terminal by the signal recognition particle (SRP) for proper translation and translocation into the endoplasmic reticulum (ER). Even though this strategy places GFP at the end of the cytosolic tail, it is commonly and successfully used to study transmembrane proteins containing cytoplasmic sorting motifs such as LAMP1 and MHC II, as well as mammalian MHC I without affecting their intracellular localization (Wubbolts et al., 1996; Falcón-Pérez et al., 2005; Wälchli et al., 2014; Dirk et al., 2016).

We expressed the selected Atlantic cod MHC I variants in MelJuSo cells (i.e., human melanoma cells). Despite not being immune cells they express peptide-loaded MHC II and all components required for MHC II antigen presentation and have previously been used as a model for antigen-presenting cells (Wubbolts et al., 1996; Paul et al., 2011). As Atlantic cod lack MHC II but have an expanded repertoire of MHC I, we chose this cell line (MelJuSo) since it expresses many immune-specific genes and proteins controlling MHC II transport in case any of the Atlantic cod MHC I variants would require similar molecules to mediate their transport. While GFP-tagged human MHC I (HLA-A2-GFP) was predominantly found at the plasma membrane, as previously reported (Wälchli et al., 2014), none of the Atlantic cod selected variants showed a cell surface localization (Figure 1B). Variants 1 and 4 localized on vesicles (Figure 1B), variants 2 and 3 had an ER-like localization, and variant 5 formed aggregates (Figure 1B). Similar results were obtained by transfecting MelJuSo cells with the same MHC I variants tagged with HA instead of GFP (Supplementary Figure S2), demonstrating that the GFP tag does not alter the intracellular localization of Atlantic cod MHC I variants. Co-transfection of variant 2, 3 or 5 together with Atlantic cod β2-microglobulin (β2M) did not alter their distribution in the cell, indicating that the localization of these variants is not a consequence of the lack of association with Atlantic cod β2M in MelJuSo cells (Supplementary Figure S3).

Object based colocalization analysis confirmed that only a small percentage (<10%) of human MHC I co-localized with lysotracker red, a fluorescent dye that stains acidic organelles (Figure 1C). In contrast, 84% of Atlantic cod MHC I variant 1% and 63% of variant 4 co-localized with lysotracker red, indicating that their main localization is to acidic compartments (Figure 1C).

To determine that the localization of Atlantic cod MHC I variant 1 to acidic compartments was not the result of its degradation into autolysosomes due to unproper protein folding, we stained MelJuSo cells transfected with Atlantic cod MHC I variant 1 for LC3, an autolysosomal marker (Supplementary Figure S4). No increased LC3 staining was observed in the transfected cells, and no overlap between LC3 and Atlantic cod MHC I variant 1-GFP. This indicates that Atlantic cod MHC I variant 1 localization to acidic compartments is not due to its clearance into autolysosomes.

MHC I variant 1 has the strongest vesicular phenotype with the highest colocalization with lysotracker red, suggesting that the hypothetical sorting motifs present in this variant could be responsible for its endolysosomal localization, as previously hypothesized (Malmstrom et al., 2013).

### Atlantic cod MHC I variant 1 cytosolic tail does not contain cytosolic sorting signals recognized by the mammalian trafficking system

From the five selected Atlantic cod MHC I variants expressed in MelJuSo cells, MHC I variant 1 has the highest colocalization with late endosomes/lysosomes. Therefore, we investigated if this protein contains any cytosolic sorting signal recognized by adaptor proteins for transport to endolysosomal compartments.

**FIGURE 2:**
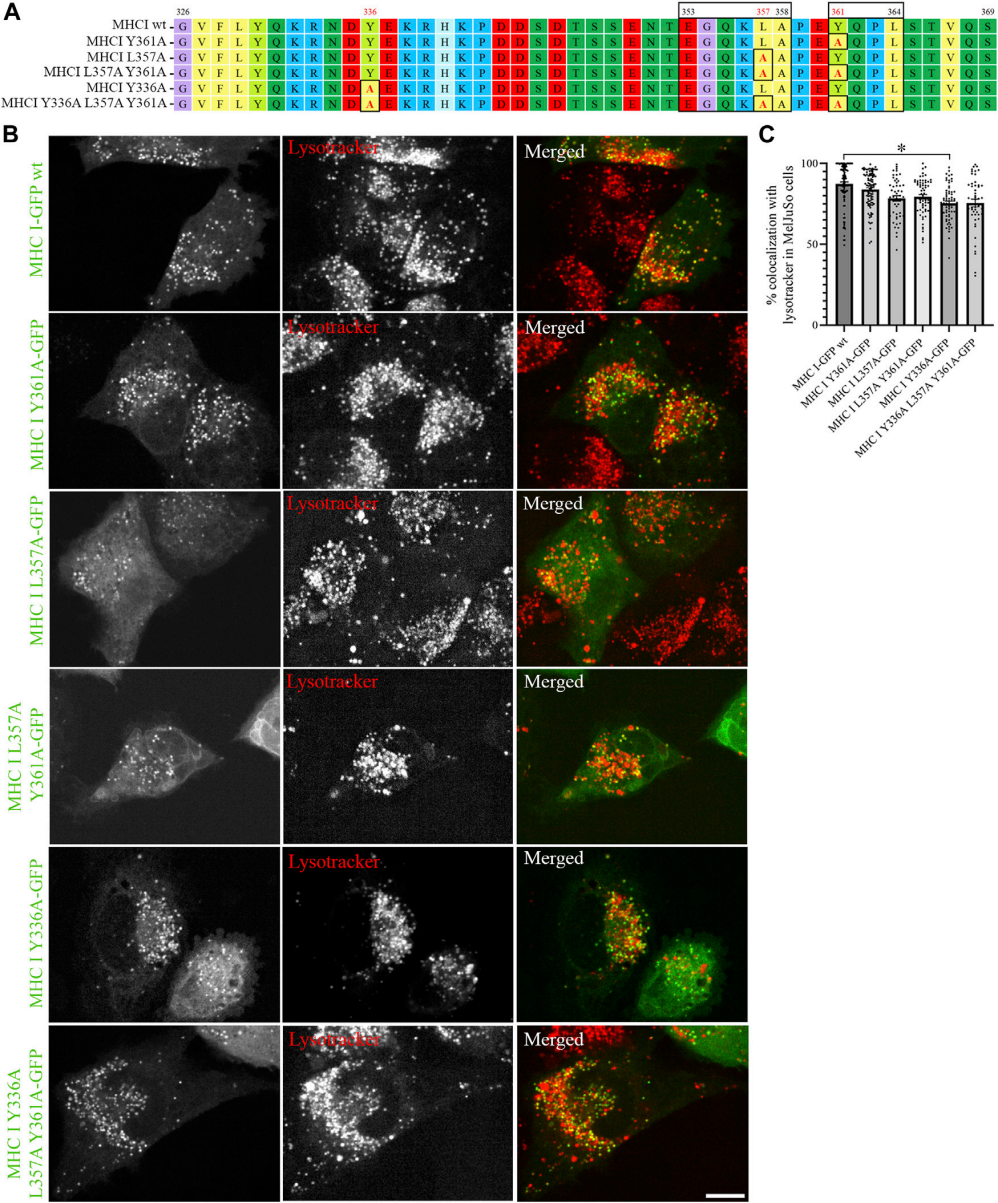
Point mutations in putative sorting motifs of Atlantic cod MHC I variant 1 cytosolic tail do not affect its intracellular localization in MelJuSO cells. **(A)** Amino acid sequences of Atlantic cod MHC I variant 1 wild type (wt) and its substitution mutants generated by introducing point mutations in hypothetical sorting motifs. All substitutions are marked with black box and red letters. Numbers above the sequences mark amino acid positions. **(B)** Representative images of MelJuSo cells transiently transfected with Atlantic cod MHC I-GFP variant 1 or the five substitution mutants (green). The cells were stained with lysotracker red and imaged using an Andor Dragonfly spinning-disk confocal microscope. Colocalization between green and red channels results in yellow signal in the merged panels. Scale bar: 10 μm. **(C)** The graph represents the percentage of colocalization between Atlantic cod MHC I variant 1 wt or the five substitution mutants and lysotracker red. Colocalization was done by object-based analysis using ImageJ software. Values represent the mean ± s.e.m. from at least three independent experiments. *n* ≥ 48 cells. *p < 0.05 (Two-tailed paired Student *t*-test).

Sequence analyses have previously suggested that the cytosolic tail of Atlantic cod MHC I variant 1 may contain two sorting motifs for endosomal trafficking: a dileucine motif (EGQKLA) in amino acid (aa) position 353–358 and a tyrosine-based signal motif (YQPL) in aa position 361–364 (Malmstrom et al., 2013) (Figure 2A). To experimentally validate that these are indeed sorting signals, we introduced point mutations in their sequences and analysed the intracellular localization of the GFP-tagged mutants by live cell imaging. The introduction of a point mutation did not affect Atlantic cod MHC I localization to lysotracker-positive compartments regardless if it was introduced in the tyrosine motif (Y361A), dileucine motif (L357A), or in both (Figure 2B). In addition, neither substitution of tyrosine in position 336 with an alanine alone nor together with the point mutations L357A and Y361A affected MHC I localization to acidic compartments (Figure 2B). Object based colocalization analysis confirmed that, even though a small but significant reduction of colocalization with lysotracker red was measured for the Y336A mutant, all the mutants retained a high degree of colocalization with lysotracker (> 70%), (Figure 2C).

**FIGURE 3:**
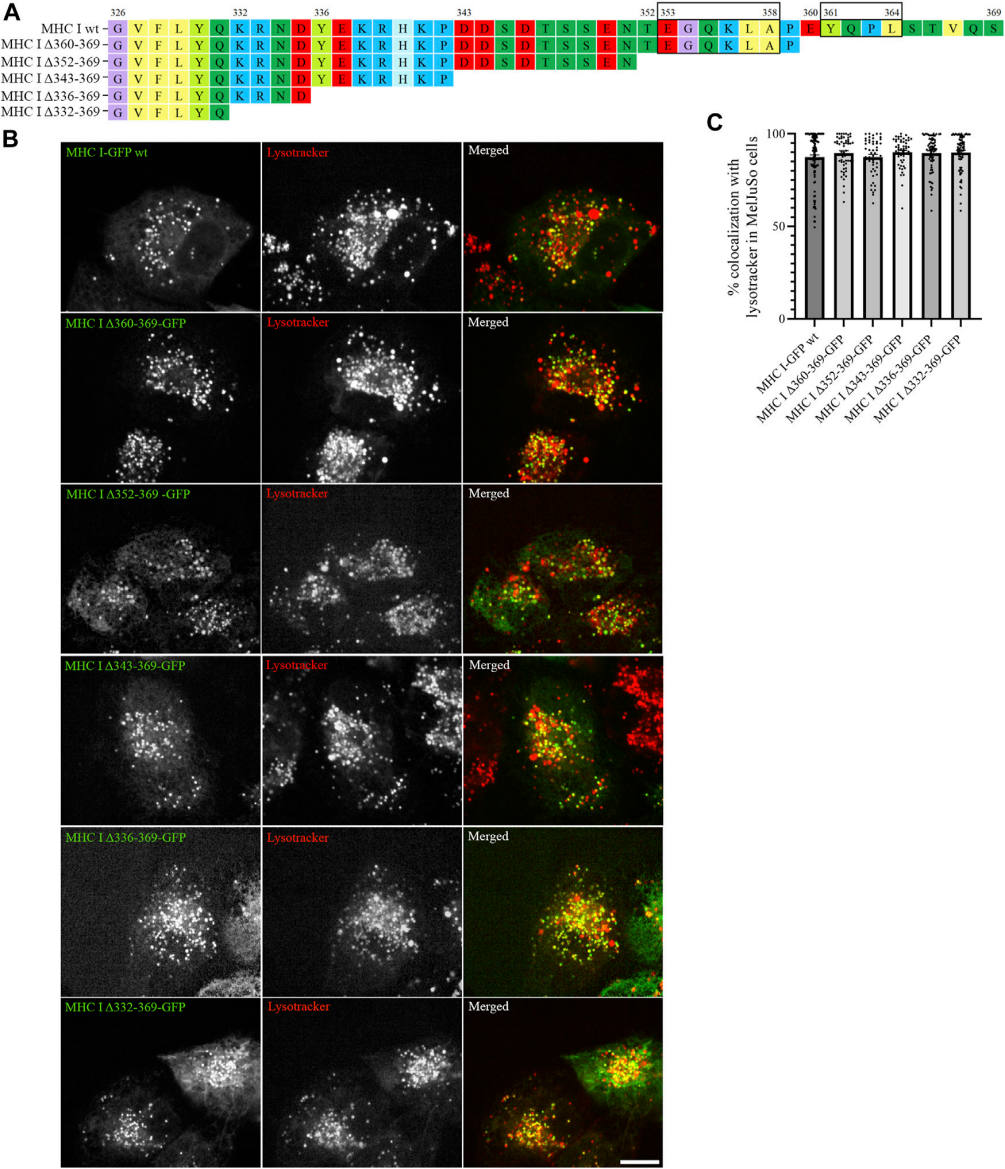
Cytosolic tail deletion mutants of Atlantic cod MHC I variant 1 do not affect its intracellular localization in MeljuSo cells. **(A)** Amino acid sequences of Atlantic cod MHC I variant 1 wt and its five deletion mutants. Putative dileucine and tyrosine sorting signals are boxed. Numbers above the sequences mark amino acid positions. **(B)** Representative images of MelJuSo cells transiently transfected with Atlantic cod MHC I-GFP variant 1 wt or the five deletion mutants (green). The cells were stained with lysotracker red and imaged using an Andor Dragonfly spinning-disk confocal microscope. Colocalization between green and red channels results in yellow signal in the merged panels. Scale bar: 10 μm. **(C)** The graph represents the percentage of colocalization between Atlantic cod MHC I variant 1 wt or the five deletion mutants and lysotracker red. Colocalization was done by object-based analysis using ImageJ software. Values represent the mean ± s.e.m. from at least three independent experiments. *n* ≥ 53 cells. Non-significant (Two-tailed paired Student *t*-test).

As the introduced point mutations did not have any major impact on MHC I localization, we next generated deletion mutants of the Atlantic cod MHC I variant 1 lacking different regions of the cytosolic tail, to investigate whether unknown sorting motifs could be present in different regions of the cytosolic tail (Figure 3A). None of the deletion mutants affected MHC I colocalization with lysotracker (Figure 3B), and no significant difference could be measured from the object based colocalization analysis (Figure 3C).

To test whether the Atlantic cod MHC I cytosolic tail contains any endosomal sorting signals, we generated a chimeric construct containing the cytosolic tail of Atlantic cod MHC I variant 1 fused with the transmembrane and luminal domain of the human lysosomal associated membrane protein LAMP1 (Supplementary Figure S5A). LAMP1 cytosolic tail contains a tyrosine sorting motif (YQTI) responsible for targeting lysosomes (Honing and Hunziker, 1995). Therefore, its replacement with Atlantic cod MHC I cytosolic tail should reveal whether this tail contains any lysosomal sorting motifs and hence sort LAMP1 to lysosomes. However, the chimeric construct re-localized to the plasma membrane in MelJuSo cells (Supplementary Figure S5B). Altogether, these results suggest that Atlantic cod MHC I variant 1 may not have any endosomal sorting signals in its cytosolic tail, or the mammalian trafficking system does not recognize them and instead uses an alternative strategy to deliver the Atlantic cod MHC I to the endolysosomal compartments. To test this, we generated a chimeric construct containing the cytosolic tail of human MHC I fused with the transmembrane and luminal domain of the Atlantic cod MHC I variant 1 (Supplementary Figure S6A). Intriguingly, the chimeric construct still localizes to acidic compartments suggesting that the intraluminal and/or transmembrane region of Atlantic cod MHC I variant 1 is responsible for its localization to endolysosomes (Supplementary Figure S6B).

To verify that Atlantic cod MHC I variant 1 localizes to acidic compartments independently of the peptide-presenting machinery in MelJuSo cells, cells were transfected with Atlantic cod MHC I variant 1 and stained for endogenous MHC II, Ii and MHC I. If Atlantic cod MHC I variant 1 would use the endogenous MHC I and/or MHC II machinery in MelJuSO cells, we would expect re-localization of these proteins. However, no such change was observed (Supplementary Figures S7A, B). Furthermore, the localization of Atlantic cod MHC I variant 1 to acidic compartments was unaffected in cells devoid of MHC II components like in MelJuSo cells knocked-out for Ii, and in U2OS cells that lack both Ii and MHC II (Supplementary Figures S7C–F).

### Atlantic cod MHC I variants localize to the endolysosomal system in Atlantic cod cells

**FIGURE 4:**
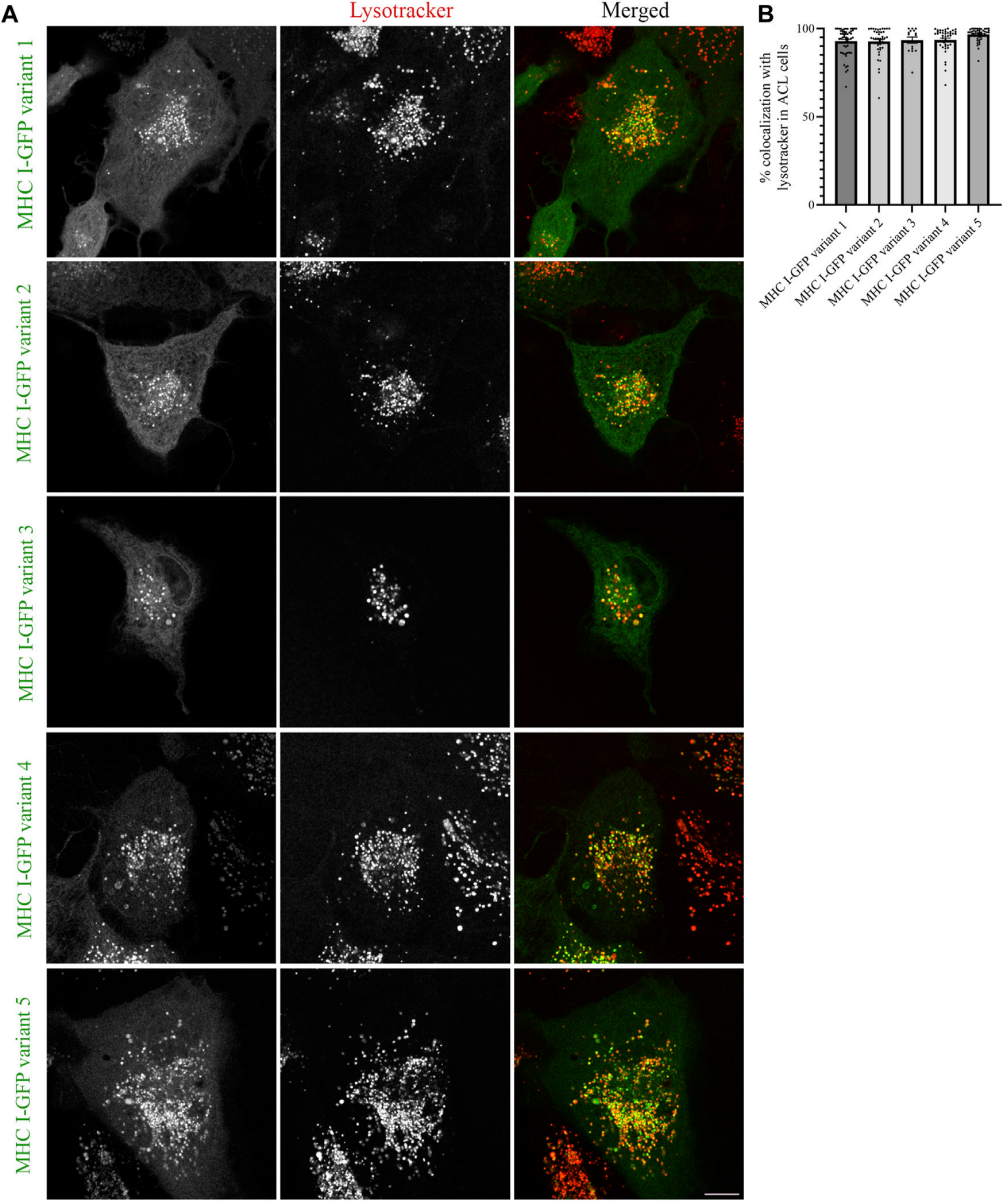
Intracellular localization of five Atlantic cod MHC I variants in ACL cells. **(A)** Representative images of ACL cells transiently transfected with five selected Atlantic cod MHC I-GFP variants (green). The cells were stained with lysotracker red and imaged using a Zeiss LSM880 Fast AiryScan microscope. Colocalization between green and red channels results in yellow signal in the merged panels. Scale bar: 10 μm. **(B)** The graph represents the percentage of colocalization between Atlantic cod MHC I variant 1–5 and Lysotracker Red. Colocalization was done by object-based analysis using ImageJ software. Values represent the mean ± s.e.m. from at least three independent experiments. *n* ≥ 15 cells. Non-significant (Two-tailed paired Student *t*-test).

The results obtained from MelJuSo cells depend on Atlantic cod MHC I associating with human homologs for proper folding and traffic at 37°C, which is not the ideal temperature for this cold-adapted organism. To exclude that the results obtained in MelJuSo cells are not affected by non-physiological temperature, we repeated the same experiments performed in MelJuSo cells in Atlantic cod larva (ACL) cells. We first expressed the five selected Atlantic cod MHC I variants in ACL cells to evaluate whether their intracellular localization was comparable to the localization observed in human MelJuSo cells. Similar to the results in MelJuSo cells, both variant 1 and 4 were present mostly in acidic compartments (Figure 4A). However, in ACL cells the other three variants also showed more than 90% colocalization with lysotracker (Figure 4B), suggesting that these variants did not express correctly in MelJuSo cells.

**FIGURE 5:**
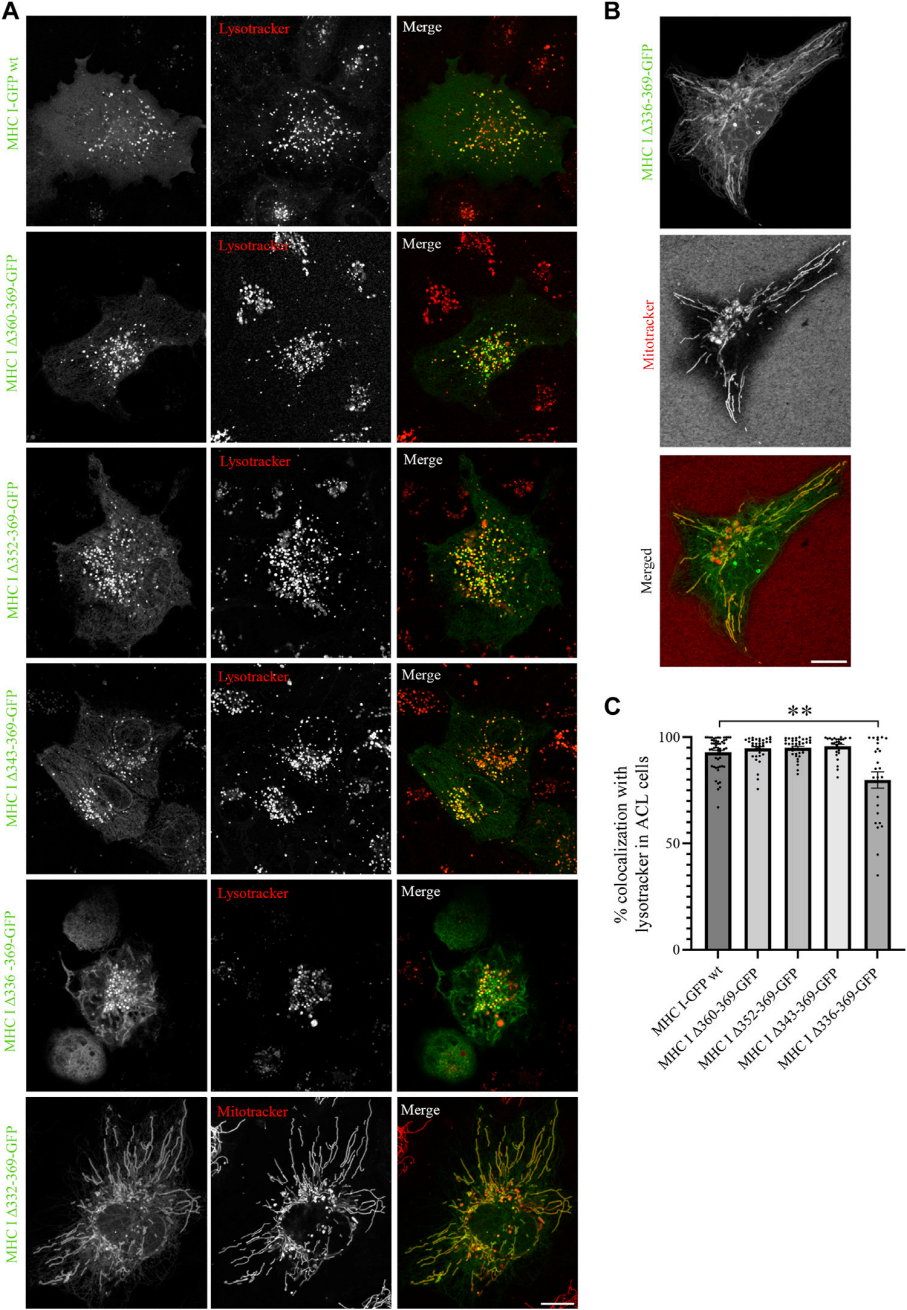
Cytosolic tail deletion mutants of Atlantic cod MHC I variant 1 relocate MHC I to mitochondria in ACL cells. **(A, B)** Representative images of ACL cells transiently transfected with Atlantic cod MHC I variant 1-GFP wt or the five deletion mutants (green). The cells were stained with either lysotracker red or mitotracker deep red and imaged using a Zeiss LSM880 Fast AiryScan microscope. Colocalization between green and red channels results in yellow signal in the merged panels. Scale bar: 10 μm. **(C)** The graph represents the percentage of colocalization between Atlantic cod MHC I variant 1 wt or the deletion mutants and lysotracker red. Colocalization was done by object-based analysis using ImageJ software. Values represent the mean ± s.e.m. from at least three independent experiments. *n* ≥ 25 cells. **p < 0.01 (Two-tailed paired Student *t*-test).

Next, we expressed the five deletion mutants of Atlantic cod MHC I variant 1 in ACL cells. Intriguingly, while Atlantic cod MHC I variant 1 deletion mutants Δ360-369, Δ352-369, and Δ343-369 still colocalized approximatively 90% with lysotracker as in MelJuSo cells (Figures 5A, C), the Δ336-369 showed a significant reduction in colocalization with acidic compartments in Atlantic cod cells, as it also localized to mitochondria (Figures 5A, B). Even more surprisingly, the MHC I deletion mutant lacking the entire cytosolic tail (Δ332-369) relocated completely to mitochondria (Figure 5A). Furthermore, even if the chimeric LAMP1-MHC I construct containing the cytosolic tail of Atlantic cod MHC I variant 1 fused with the transmembrane and luminal domains of human LAMP1 was able to partially reach the endolysosomal compartments in Atlantic cod cells, it also clearly relocated to the plasma membrane (Supplementary Figure S5C). All together, these results indicate that Atlantic cod MHC I may contain a signal motif in its cytosolic tail required for its localization to acidic compartments that is specific for Atlantic cod and that this signal is present between aa 332 and 343. To test this, we mutated tyrosine at position 336 to alanine and expressed the Y336A mutant in ACL cells. However, this mutant still localized to endolysosomal compartments (Supplementary Figures S8A, B), indicating that the tyrosine in position 336 is not part of a sorting signal responsible for the intracellular localization of Atlantic cod MHC I variant 1.

A closer analysis of the amino acid sequence between aa 332 and 343 of MHC I variant 1 revealed the presence of positively charged KR amino acid residues. These residues are also present in the other four variants selected in this study, and have previously been suggested to represent non-canonical sorting signals for lysosomal targeting (Li et al., 2015). To examine whether the lysosomal localization of Atlantic cod MHC I is due to the non-canonical KR sorting motif, we replaced these two residues with alanine in the full-length Atlantic cod MHC I variant 1 and variant 1 Δ336-369. However, the mutations in the putative KR sorting motif did not change the location of MHC I to lysotracker-positive compartments (Supplementary Figures S8C, D), hence the KR motif is not part of a sorting motif in Atlantic cod MHC I variant 1. Because we could not determine any sorting motifs in the region between aa 332 and 342, the change in location for deletion mutant Δ336-369 and Δ332-369 is most likely the result of improper folding and conformation of the protein.

Altogether these results indicate that MHC I variant 1 does not have any classic sorting motifs in its cytosolic region. To further investigate whether the sorting of this MHC I variant to endolysosomes is independent of its cytosolic tail, we expressed the chimeric construct containing the cytosolic tail of human MHC I fused with the transmembrane and luminal domain of Atlantic cod MHC I variant 1 in ACL cells (Supplementary Figures S6A, C). Intriguingly, while the human MHC I is retained in the ER in Atlantic cod cells, the chimeric construct localizes to acidic compartments, further supporting that Atlantic cod cytosolic tail does not contain lysosomal sorting motifs.

### Atlantic cod MHC I variant 1 interacts with tapasin in the endocytic pathway

**FIGURE 6:**
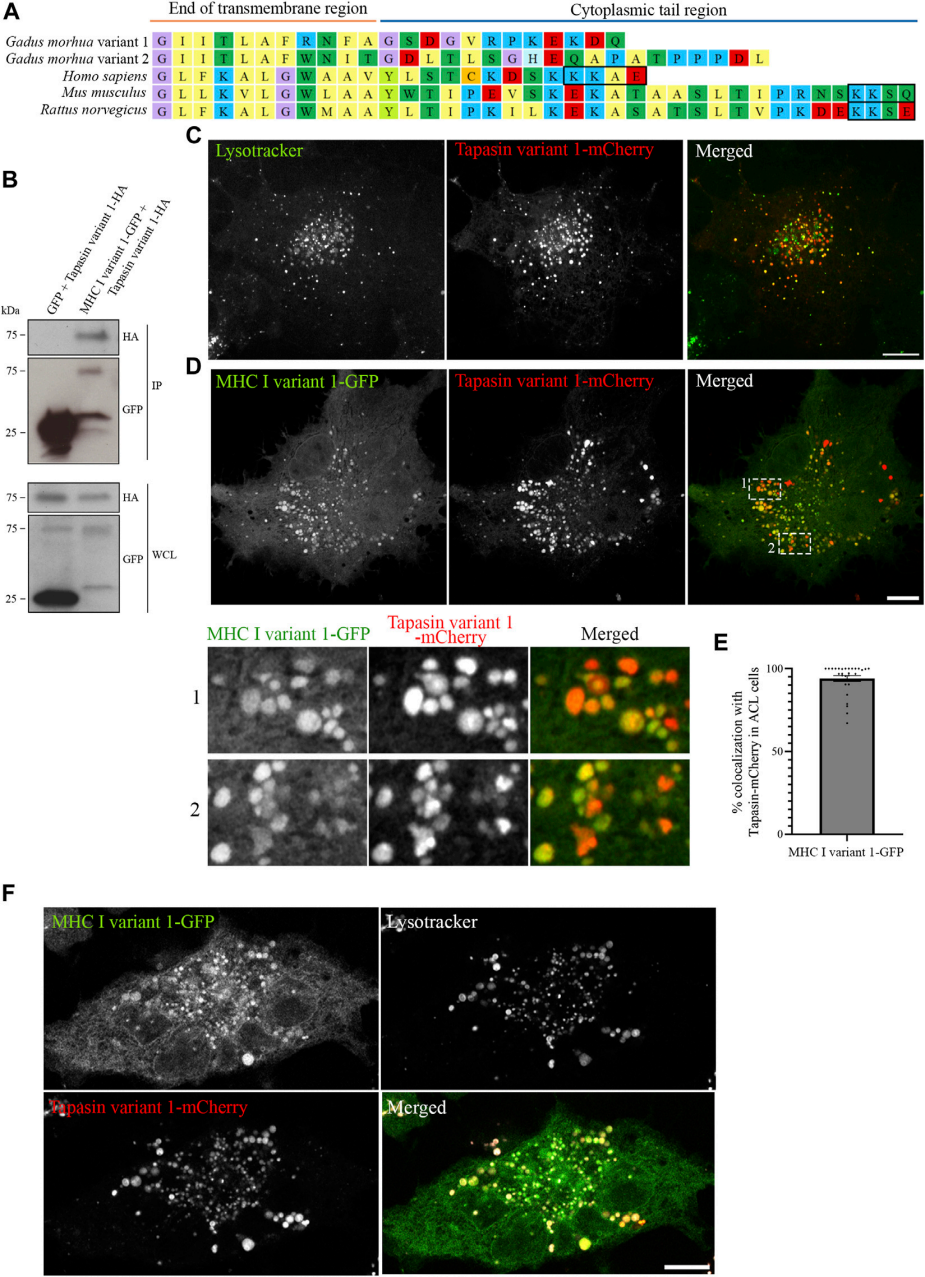
Atlantic cod MHC I variant 1 interacts with tapasin and colocalize on endolysosomes. **(A)** Amino acid sequences of the cytosolic tails of tapasin from Atlantic cod (*Gadus morhua*), human (*Homo sapiens*), mouse (*Mus musculus*) and rat (*Rattus norvegicus*). Sequences were manually curated and aligned with MEGA7. ER retention signals (KKXX) are boxed. **(B)** ACL cells were transiently co-transfected with Tapasin variant 1-HA and either GFP or MHC I variant 1-GFP, lysed and subjected to IP with GFP magnetic agarose beads. Whole-cell lysates (WCL) and immunoprecipitates (IP) were subjected to Western blot analysis using the indicated antibodies. **(C)** Representative image of an ACL cell transiently transfected with Atlantic cod Tapasin variant 1-mCherry (red). Cells were stained with green lysotracker and imaged using a Zeiss LSM880 Fast AiryScan microscope. Colocalization between green and red channels results in yellow signal in the merged panels. Scale bar: 10 μm. **(D)** Representative image of an ACL cell transiently co-transfected with Atlantic cod MHC I variant 1-GFP (green) and Tapasin variant 1-mCherry (red). The cells were imaged using a Zeiss LSM880 Fast AiryScan microscope. Colocalization between green and red channels results in yellow signal in the merged panels. Scale bar: 10 μm. Magnification of boxed areas show colocalization between MHC I variant 1-GFP and Tapasin variant 1-mCherry. **(E)** The graph represents the percentage of colocalization between Atlantic cod MHC I variant 1-GFP and Tapasin variant 1-mCherry. Colocalization was done by object-based analysis using ImageJ software. Values represent the mean ± s.e.m. from at least three independent experiments. *n* = 28 cells. **(F)** Representative image of an ACL cell transiently co-transfected with Atlantic cod MHC I variant 1-GFP (green) and Tapasin variant 1-mCherry (red). The cells were stained with lysotracker deep red (gray) and imaged using a Zeiss LSM880 Fast AiryScan microscope. Colocalization between green and red channels results in yellow signal in the merged panels. Scale bar: 10 μm.

**FIGURE 7:**
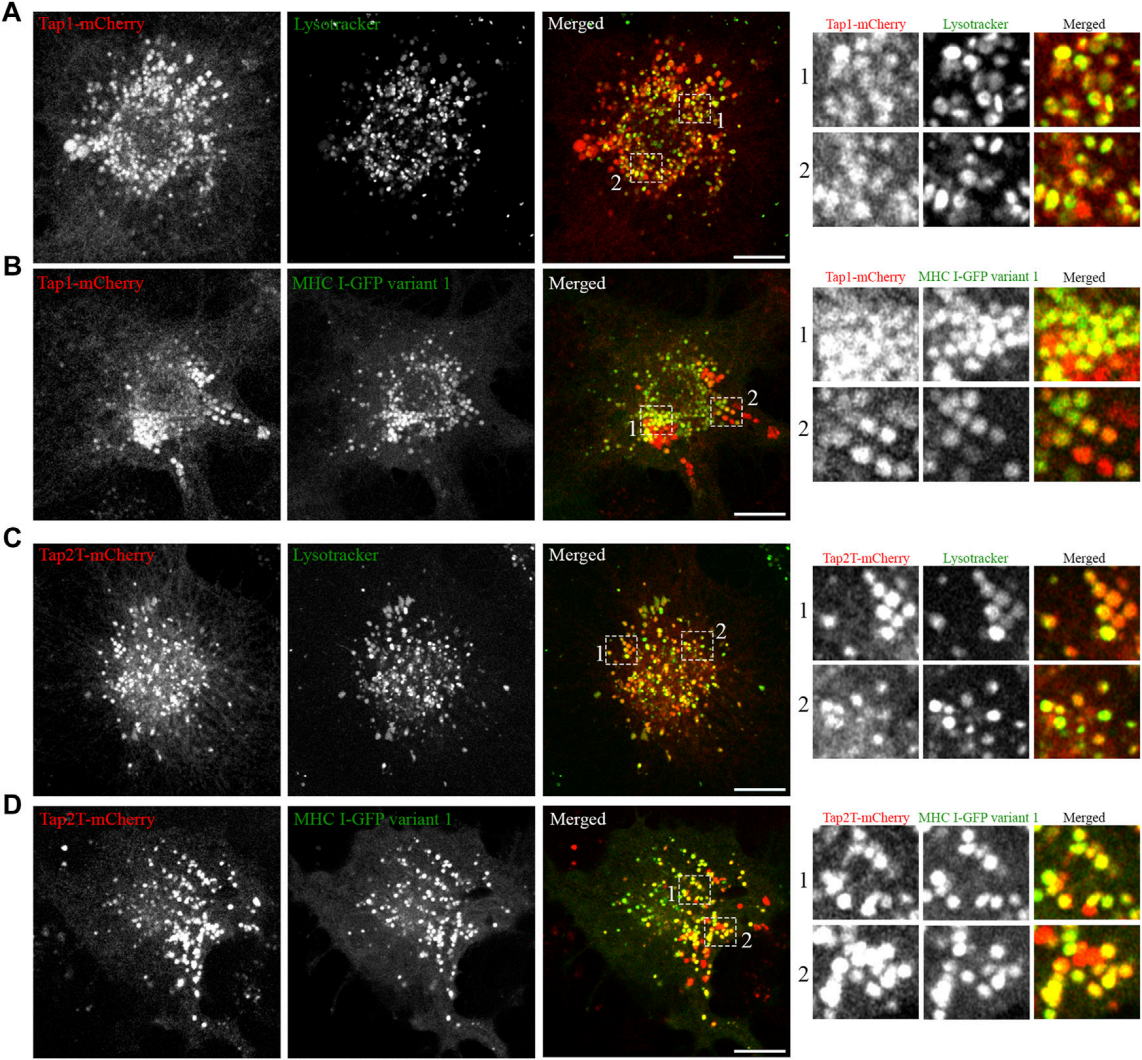
Atlantic cod PLC components Tap1 and Tap2T colocalize with MHC I variant 1 on endosomes. **(A)** Representative image of an ACL cell transiently transfected with cod Tap1-mCherry (red), stained with green lysotracker and imaged using a Zeiss LSM880 Fast AiryScan microscope. Colocalization between green and red channels results in yellow signal in the merged panels. Scale bar: 10 μm. Magnification of boxed areas show colocalization between Tap1-mCherry and green lysotracker. **(B)** Representative image of an ACL cell transiently co-transfected with Atlantic cod Tap1-mCherry (red) and MHC I variant 1-GFP (green) imaged using a Zeiss LSM880 Fast AiryScan microscope. Colocalization between green and red channels results in yellow signal in the merged panels. Scale bar: 10 μm. Magnification of boxed areas show colocalization between Tap1-mCherry and MHC I variant 1-GFP. **(C)** Representative image of an ACL cell transiently transfected with cod Tap2T-mCherry (red), stained with green lysotracker and imaged using a Zeiss LSM880 Fast AiryScan microscope. Colocalization between green and red channels results in yellow signal in the merged panels. Scale bar: 10 μm. Magnification of boxed areas show colocalization between Tap2T-mCherry and green lysotracker. **(D)** Representative image of an ACL cell transiently co-transfected with Atlantic cod Tap2T-mCherry (red) and MHC I variant 1-GFP (green) imaged using a Zeiss LSM880 Fast AiryScan microscope. Colocalization between green and red channels results in yellow signal in the merged panels. Scale bar: 10 μm. Magnification of boxed areas show colocalization between Tap2T-mCherry and MHC I variant 1-GFP.

Having established that all five Atlantic cod MHC I variants analysed in this study localize to the endolysosomal system in Atlantic cod cells, we next addressed whether the endolysosomal system could be a possible site for antigen loading onto MHC I in Atlantic cod. In mammals, tapasin is an ER resident protein and a MHC I-specific chaperone that helps with initial loading of peptides onto MHC class I molecules as part of the peptide loading complex (PLC) (Sadasivan et al., 1996; Li et al., 1997; Ortmann et al., 1997; Rizvi and Raghavan, 2010; Blees et al., 2017; Wieczorek et al., 2017). In mammals, tapasin is retained in the ER due to a double lysine motif (KKXX) located C-terminally (Roder et al., 2011). Intriguingly, Atlantic cod have two gene variants of tapasin, which differ from mammalian tapasin and lack the KKXX motif for ER-retention (Figure 6A). Therefore, we were interested in whether Atlantic cod tapasin could be involved in peptide loading outside the ER supporting a mechanism for cross presentation. First, we evaluated whether tapasin interacts with MHC I in Atlantic cod. ACL cells where transiently co-transfected with Atlantic cod tapasin-HA and either GFP-tagged MHC I variant 1 or GFP as negative control. Immunoprecipitation experiments using GFP magnetic beads confirmed that GFP-tagged Atlantic cod MHC I variant 1, but not GFP, interacts with tapasin (Figure 6B). To further investigate whether tapasin localizes to the ER as in mammals, we analyzed GFP-tagged MHC I variant 1 and tapasin-mCherry localization in ACL cells. Surprisingly, in Atlantic cod tapasin colocalizes with MHC I in the endolysosomal system (Figures 6C–F), suggesting this could be the site where peptide loading occurs and that tapasin may have a role in peptide loading and editing in this compartment. As tapasin is only one of many proteins involved in the peptide loading complex (PLC), we investigated whether other PLC proteins are localized to the endolysosomal system. Interestingly, both Atlantic cod Tap1 and Tap2T co-localize with Atlantic cod MHC I on endolysosomes (Figure 7).

In summary, our results open the possibility that in Atlantic cod both MHC I and PLC proteins function in a different compartment than their mammalian homologues. The development of specific tools to study the Atlantic cod immune system is needed to verify whether the endolysosomal system is the major site for antigen loading in Atlantic cod.

## Discussion

In this study, we have demonstrated for the first time that Atlantic cod MHC I variants travel to the endolysosomal system in an Atlantic cod cell-line system and that this is independent of cytosolic sorting signals. This is intriguing as MHC I is known to primarily localize to the plasma membrane in mammals (Donaldson and Williams, 2009; Fritzsche and Springer, 2013; Ruggiero and Springer, 2022). Considering the large variability in amino acid composition and length of the cytosolic domain of the selected Atlantic cod MHC I variants (Figure 1A), it was surprising to observe that they all sorted to acidic compartments in ACL cells. This suggests that MHC I molecules in Atlantic cod operates differently than in mammals. One hypothesis is that Atlantic cod MHC I variants contain atypical sorting motifs for endolysosomal trafficking, which differ from classical dileucine- and tyrosine-based sorting motifs present in mammals (Bakke and Nordeng, 1999; Bonifacino and Traub, 2003; Lizée et al., 2005; Basha et al., 2008). A second hypothesis is that no specific sorting motif is present in the cytosolic domain, as indicated by the large sequence variability in the cytosolic domain of the different Atlantic cod MHC I variants, and that transport to the endolysosomal system could rely on a different mechanism.

Our results are in line with the second hypothesis, indicating a different mechanism not relying on sorting motifs in the cytosolic domain. Indeed, neither point mutations nor deletions of the cytosolic tail resulted in relocation of Atlantic cod MHC I variant 1, suggesting that no sorting motifs are present in the cytosolic tail of this variant. Furthermore, replacement of the Atlantic cod MHC I cytosolic tail with the corresponding human region retains the Atlantic cod MHC I to acidic compartments (Supplementary Figure S6). Even though we observed a re-location of deletion mutants MHC I variant 1 Δ336-369 and Δ332-369 to the mitochondria in ACL cells (Figure 5), it is likely to be the result of altered protein folding and membrane insertion rather than removal of sorting motifs. In line with this, the introduction of point mutations in the amino acid region 332–343 did not affect the endolysosomal localization of MHC variant 1, suggesting the absence of sorting motifs (Supplementary Figure S8). Transmembrane proteins require charged residues in their cytosolic region for proper membrane insertion, and removing these sequences as done for deletion mutants such as MHC I variant 1 Δ336-369 and Δ332-369 can result in improper membrane insertion (von Heijne, 1989; von Heijne, 1992; von Heijne, 2006).

Our results indicate that Atlantic cod MHC I variant 1 does not contain any functional dileucine- and tyrosine-based sorting motifs in its cytosolic tail. Interestingly, while Malmstrøm et al., 2013 hypothesized that the EGQKLA signal found in Atlantic cod MHC I variant 1 cytosolic tail is a potential dileucine-like signal, Frenette et al., 2021 recently rejected this possibility due to the absence of the last leucine in the hypothetical dileucine sorting motif (Bonifacino and Traub, 2003). Even though the latter work is based on another species, namely haddock (*Melanogrammus aeglefinus*) (Frenette et al., 2021), haddock and Atlantic cod have some important similarities. They are both members of the gadiform lineage within the same family Gadidae. Furthermore, they do not have a functional MHC class II pathway, and both species have an expanded MHC class I repertoire (Malmstrom et al., 2016; Tørresen et al., 2018). Similar to Atlantic cod, haddock has a dileucine-like sorting motif (EGQNLA) in its MHC I cytosolic tail that deviates from a typical dileucine signal by lacking the last leucine (Bonifacino and Traub, 2003). However, no tyrosine-like signal is present in the haddock MHC I variants (Frenette et al., 2021). Our findings that the cytosolic tail of Atlantic cod MHC I variant 1 does not contain any sorting motifs are therefore in line with the speculation from Frenette et al. (2021) that the dileucine-like motif is not a sorting signal.

It is interesting to note that Atlantic cod MHC I variants 1 and 4 are sorted to the endolysosomal system in both human and Atlantic cod cells (Figures 1B, 4A). This might indicate that Atlantic cod utilize, at least for these two variants, a conserved trafficking mechanisms present in mammals. The ER retention observed for Atlantic cod MHC I variant 2, 3 and aggregation of variant 5 in MelJuSo cells may be the result of protein temperature sensitivity. Atlantic cod is a cold-adapted organism, and ACL cells grow at 15°C. Expression of Atlantic cod proteins in mammalian cells grown at 37°C might therefore result in improper protein folding. Moreover, Atlantic cod MHC I variant 5 is part of the non-classical MHC I Z lineage that does not exist in mammals, and it is likely that the mammalian system cannot recognize it, thus forming aggregates. Besides this, Atlantic cod MHC I variants might not be recognized properly by the human trafficking system due to differences in adaptor proteins. We performed alignment analysis between human and Atlantic cod adaptor proteins GGAs and APs using their overall sequence (Supplementary Table S1) and phylogenetic clustering (Supplementary Figures S9, 10). These results demonstrate a high degree of conserveness, indicating that the human system could potentially recognize Atlantic cod sorting signals. Non-etheless, the alignment analysis revealed some length variation and regions with more variation between human and Atlantic cod adaptors, in addition to potentially more GGA copies in teleosts compared to mammals. These differences, as well as protein adaptation to temperature, might explain why some of the selected Atlantic cod MHC I variants did not localize to the same compartments in MelJuSo cells.

Our data demonstrate that Atlantic cod tapasin variants do not contain any ER-retention signals and that Atlantic cod tapasin variant 1 has a surprisingly strong endolysosomal localization in ACL cells (Figures 6A, C). Furthermore, Atlantic cod MHC I variant 1 and tapasin variant 1 interacts and colocalize on endolysosomes (Figures 6B, D–F). While the interaction between tapasin and MHC I in Atlantic cod is not unexpected, as it also occurs in mammals (Rizvi and Raghavan, 2010; Blees et al., 2017), it is surprising that in Atlantic cod tapasin localizes to the endolysosomal system rather than to the ER as in mammals (Sadasivan et al., 1996; Solheim et al., 1997; Rizvi and Raghavan, 2010). This is intriguing as it might indicate that the function of Atlantic cod tapasin changed or followed a different evolutionary path. From our results, it is tempting to speculate that Atlantic cod tapasin might be important for peptide loading of Atlantic cod MHC I in the endosomal system. The localization of other Atlantic cod PLC proteins, such as Tap1 and Tap2T, to endolysosomal compartments (Figure 7) further supports this hypothesis.

Nevertheless, how do Atlantic cod MHC I molecules reach the endolysosomal compartments? One possibility is that a chaperone-like protein is responsible for the localization of MHC I. This putative chaperone may for example contain a sorting signal in its cytosolic tail while binding to the intraluminal region of MHC I, such as Ii for MHC II (Neefjes et al., 2011). However, as functional studies on Atlantic cod immunity are still in their infancy, further research is needed to reveal whether, and if so, which chaperone-molecule is needed for the transport of Atlantic cod MHC I to the endolysosomal system.

Even though there are still many unresolved questions regarding the Atlantic cod immune system, we have in this study done pioneer work to reveal some functional aspects of Atlantic cod MHC I. Most importantly, we have demonstrated that there are indeed differences between mammalian and Atlantic cod MHC I trafficking. First, Atlantic cod MHC I sorts to the endosomal system, and MHC I variant 1 does this independently of sorting motifs, while in mammals MHC I molecules are primarily located in the plasma membrane (Donaldson and Williams, 2009). Second, Atlantic cod tapasin does not localize to the ER-membrane as mammalian tapasin, but colocalizes with MHC I in the endosomal system. These differences are remarkable, as they might indicate that mammals and Atlantic cod have developed different intracellular strategies for MHC class I presentation, possibly related to the absence of the MHC class II pathway in Atlantic cod.

## Materials and methods

### Cell culture

MelJuSo wt and MeljuSo KO (Margiotta et al., 2020) cells were grown in Iscove’s Modified Dulbecco’s Medium (IMDM, Gibco) and U2OS cells were grown in Dulbecco’s modified Eaglés medium (DMEM; Lonza, BioWhittaker). Both IMDM and DMEM were supplemented with 10% fetal calf serum (FCS), 2 mM L-glutamine, 100 U/mL penicillin, 100 μg/mL streptomycin. MelJuSo and U2OS cells were maintained in a 5% CO_2_ atmosphere at 37°C. ACL cells (Jensen et al., 2013) were grown in IMDM (Gibco) supplemented with 10% FCS, 2% HEPES buffer solution (Gibco), 1% L-glutamine (Gibco), 1% Non-Essential Amino Acids solution (Gibco) and 0.1% penicillin and streptomycin. Cells were maintained in a 4.5% CO_2_ atmosphere at 15°C.

### Constructs and site-directed mutagenesis

pEGFP-N3 and pcDNA3.1-C-HA constructs containing Atlantic cod MHC I variants, the chimeric human LAMP1- Atlantic cod MHC I and the Atlantic cod-human MHC I chimera were purchased from GenScript. cDNA sequences from Atlantic cod MHC I U lineage were downloaded from NCBI after a text search ensuring that the sequences from Malmstrøm et al. (Malmstrom et al., 2013) were included. Sequences without a cytoplasmic tail were excluded. The remaining sequences were annotated according to the tail content e.g., long, short, one or both signals reported in Malmstrom et al. (2013). MHC I Z lineage representatives were found using NCBI tblastn. Reference MHC I sequences were added from Grimholt et al., 2015 together with predicted alpha 1, 2 and 3 regions from the latest Atlantic cod genome assemblies GadMor2/GadMor3 (https://www.ncbi.nlm.nih.gov/assembly/GCF_902167405.1/) (Tørresen et al., 2017; Matschiner et al., 2022) and the Celtic Sea population representative (Kirubakaran et al., 2020). All sequences were aligned using MEGA7 and MUSCLE. A tree was inferred from the translated protein alignment using the neighbour joining method, Poisson distribution and 500 bootstrap replicates. The tree roughly resolved into several subclades of MHCI U lineage, but with little support. From this tree, four MHC I U sequences and one MHC I Z sequence were selected to collectively represent some of the most common tail configurations in terms of length, signals present and other key amino acids of interest. Variant 1 is a combination of two cDNA entries: AF414217.1 has the leading part of AF414205.1. AF414217.1 contains both signals in its tail, but also a slightly different transmembrane region configuration. However, this configuration was not found in any complete cDNA clone. Thus, the leading region of AF414205.1 was fused onto AF414217.1. Variant 2 corresponds to KF033024.1, Variant 3 to KF032939.1, Variant 4 is KF032993.1 and Variant 5 is XM_030346880.1. All mutants were obtained using the Quick Change II XL Site-Directed Mutagenesis Kit from Agilent Technologies using the templates and primers listed in Supplementary Table S3. Primers were designed using QuickChange Primer Design (agilent.com) and ordered from Eurofins Genomics. We used the protocol provided by the kit and used 10 min extension time and 30 ng of dsDNA template. mCherry-pcDNA3.1-C-HA Atlantic cod tapasin variant 1 was purchased from GenScript and corresponds to XM_030346628.1. mCherry-pcDNA3.1-C-HA Atlantic cod Tap1 and Tap2T were purchased from GenScript and were manually extracted from assembly https://doi.org/10.6084/m9.figshare.8215994.v1. pCIpA102-G-HLA-A2-GFP was provided by Tone Fredsvik Gregers & Sébastien Walchli (Addgene plasmid #85162; http://n2t.net/addgene:85162; RRID:Addgene_85162). pEGFP-Rab7a was provided by Cecilia Bucci (University of Salento, Italy) (Bucci et al., 2000).

### Sequence alignments and phylogenetic tree of adaptor proteins

Sequences of human GGAs and APs were obtained from https://vertebrate.genenames.org <https://vertebrate.genenames.org/> through their link to Refseq gene models. All canonical transcripts were downloaded through NCBI. For Atlantic cod, GGAs and APs were obtained through gene name searches (https://www.ncbi.nlm.nih.gov/data-hub/gene/taxon/8049/). In addition, a small set of protein coding sequences from other species were downloaded from GenBank to be included in a multiple protein sequence alignment. Atlantic cod and human transcript sequences were curated using MEGA7 and aligned using muscle. As proteins, pair-wise p-distances were calculated using MEGA7. The p-distance reflects the proportion of amino acid sites at which two sequences compared are different. Using the translated protein alignment, additional protein sequences were added and re-aligned using muscle. A simple unrooted neighbour-joining tree with Poisson distribution and 500 bootstrap replicates was generated to ensure that the annotated Atlantic cod gene models likely share ancestry with the annotated human gene models. With the exception of one AP protein annotated as AP1B that belongs to AP2B in Atlantic cod, all annotations clustered as expected.

### Antibodies and reagents

Primary antibodies used in this study for western blot (WB) and immunofluorescence (IF) analysis include anti-GFP (Abcam, ab6556, WB 1:1,000), anti-HA (Abcam, ab9110, WB 1:100, IF 1:300), anti-Myc (Abcam, ab32 [9E10], IF 1:200), anti-MHC I (HLA-A/B/C, Santa Cruz, sc-52810, IF 1:200), anti-Ii (CD74, BD biosciences, 555538, WB 1:1,000, IF 1:500), anti MHC II (HLA-DR, Abcam, ab-20181, WB 1:1,000, IF 1:500), anti-LC3 (MBL, PM036, IF 1:500) and anti-tubulin (Life Technologies, 13–8,000, WB 1:24000). Alexa Fluor secondary antibodies (Invitrogen) were used for IF at dilution 1:200 and secondary antibodies conjugated to horseradish peroxidase for immunoblotting studies (GE Healthcare) were diluted 1:5,000. For nuclear staining, DAPI (Sigma-Aldrich) was used at 0.1 μg/mL. LysoTracker^™^ Red DND-99 (Invitrogen), LysoTracker^™^ Green DND-26 (Invitrogen) and MitoTracker^™^ Deep Read FM (Invitrogen) were used at a final concentration of 50 nM.

### Immunoblotting

Cells were lysed in lysis buffer (125 mM K-acetate, 25 mM Hepes, 5 mM EGTA, and 2.5 mM Mg-acetate, pH 7.2) complemented with 0.5% NP-40, protease inhibitor cocktail, and DTT. Cell lysates were subjected to SDS-PAGE followed by blotting onto polyvinylidene fluoride (PVDF) membranes (Millipore) and incubation with primary antibodies diluted in 2% blotting grade non-fat dry milk (BioRad). Next, the membranes were incubated with secondary antibodies conjugated to horseradish peroxidase (HRP) (GE Healthcare). For chemiluminescence detection, ECL Prime Western Blotting Detection (GE Healthcare) was used followed by imaging using a ChemiDoc imaging system from.Bio-Rad.

### Transfection

For transient transfection of MelJuSo and U2OS cells, Lipofectamine 2000 (Life Technologies) was used according to the manufacturer’s protocol. The cells were transfected 24 h prior to further execution of experiments at a confluency of approximately 50%–70%. ACL cells were transfected by using Amaxa nucleofector 2b (Lonza) program X-005. For each condition, 50% of the cells from a confluent T25 flask (VWR^®^) was transfected with 2 µg of DNA and seeded out directly onto 35-mm-diameter imaging dish with glass bottom (MatTek) coated with 20 μg/μL fibronectin (F2006-1MG Sigma). The ACL cells were transfected at least 4 days prior to further execution of experiments.

### Co-immunoprecipitation

The GFP-Trap^®^_MA (Chromotek) was used for co-immunoprecipitation experiments according to the producer’s protocol. Briefly, cells transiently transfected with GFP-fusion proteins were lysed in lysis buffer (10 mM Tris-HCl pH 7.5, 150 mM NaCl, 0.5 mM EDTA, and 0.1% NP-40) and the lysates incubated for 1 h at 4°C with magnetic beads coupled to anti-GFP. The immunoprecipitated samples and their respective total lysates were loaded on SDS-PAGE gels and analyzed by western blotting.

### Immunofluorescence

MelJuSo cells grown on coverslips were fixed with 3% paraformaldehyde (PFA) for 15 min, quenched with 50 mM NH_4_Cl, then permeabilized with 0.1% Triton X-100 for 10 min, before incubation with primary antibody in 1× PBS against HA for 1 h. The cells were then incubated with Alexa Fluor 594-conjugated secondary antibody in 1× PBS for 45 min before mounting with Mowiol. The samples were visualized using the Olympus FluoView 1000 IX81 confocal laser scanning microscope (inverted) with a 60x PlanApo NA 1.35 objective.

### Live-cell microscopy

MelJuSo cells were seeded on 35-mm-diameter imaging dishes with glass bottoms (MatTek) and transfected 24 h prior to the experiment. During imaging, the MelJuSo cells were kept at 37°C and 5% CO_2_. To label acidic compartments, MelJuSo cells were incubated with LysoTracker^™^ Red DND-99 for 30 min at 37°C and 5% CO_2_ before being washed with 1x PBS and imaged, using a Zeiss LSM880 microscope equipped with a 63 × oil Plan Apo NA 1 objective. ACL cells were seeded on MatTek glass-bottom dishes coated with 20 μg/μL fibronectin (F2006-1MG Sigma) and transfected at least 4 days prior to the experiment. During imaging, the cells were kept at room temperature and 4.5% CO_2_. To label acidic compartments, ACL cells were incubated with LysoTracker^™^ Red DND-99 for 1 h at 15°C and 4.5% CO_2_ and then imaged using a Zeiss LSM880 microscope equipped with a 63 × oil Plan Apo NA 1 objective. Mitochondrial staining was performed similarly, using MitoTracker Deep Red FM.

### Image processing and analysis

Image analysis and processing was performed using ImageJ (National Institutes of Health) and Adobe Photoshop (Adobe Systems). Graphs were generated using the software GraphPad Prism 9 (GraphPad Software Inc., https://www.graphpad.com). Object-based colocalization analysis was performed using ImageJ software to quantify the degree of colocalization between MHC I vesicles and lysotracker or tapasin. Specifically, the area of MHC I with an overlapping signal from lysotracker or tapasin was measure with respect to the total area of MHC I within each cell. Statistical analysis was done using two-tailed paired Student *t*-test in Excel (Microsoft). In the figures, statistical significance is indicated as follows: *p < 0.05, **p < 0.01, ***p < 0.001.

## Data Availability

The original contributions presented in the study are included in the article/Supplementary Material, further inquiries can be directed to the corresponding author.
