# Supplementary figures and images for: Atlantic cod (Gadus morhua) MHC I localizes to endolysosomal compartments independently of cytosolic sorting signals
Source: Front Cell Dev Biol. 2023 Jan 25;11:1050323. doi: 10.3389/fcell.2023.1050323 (PMC9905690; doi:10.3389/fcell.2023.1050323)

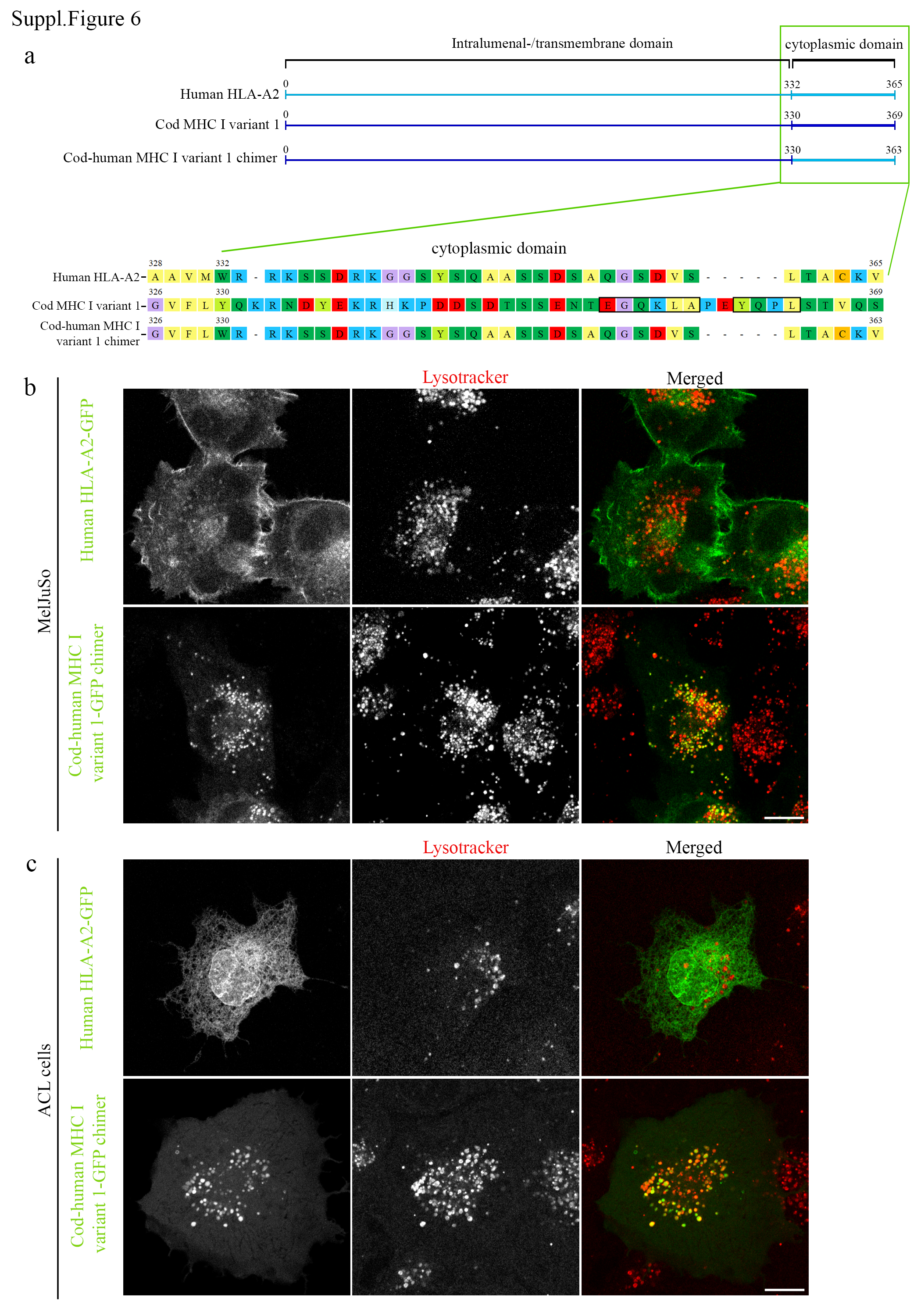

Supplement: Supplementary file 4 [file Image6.tif]

Suppl. Figure 9

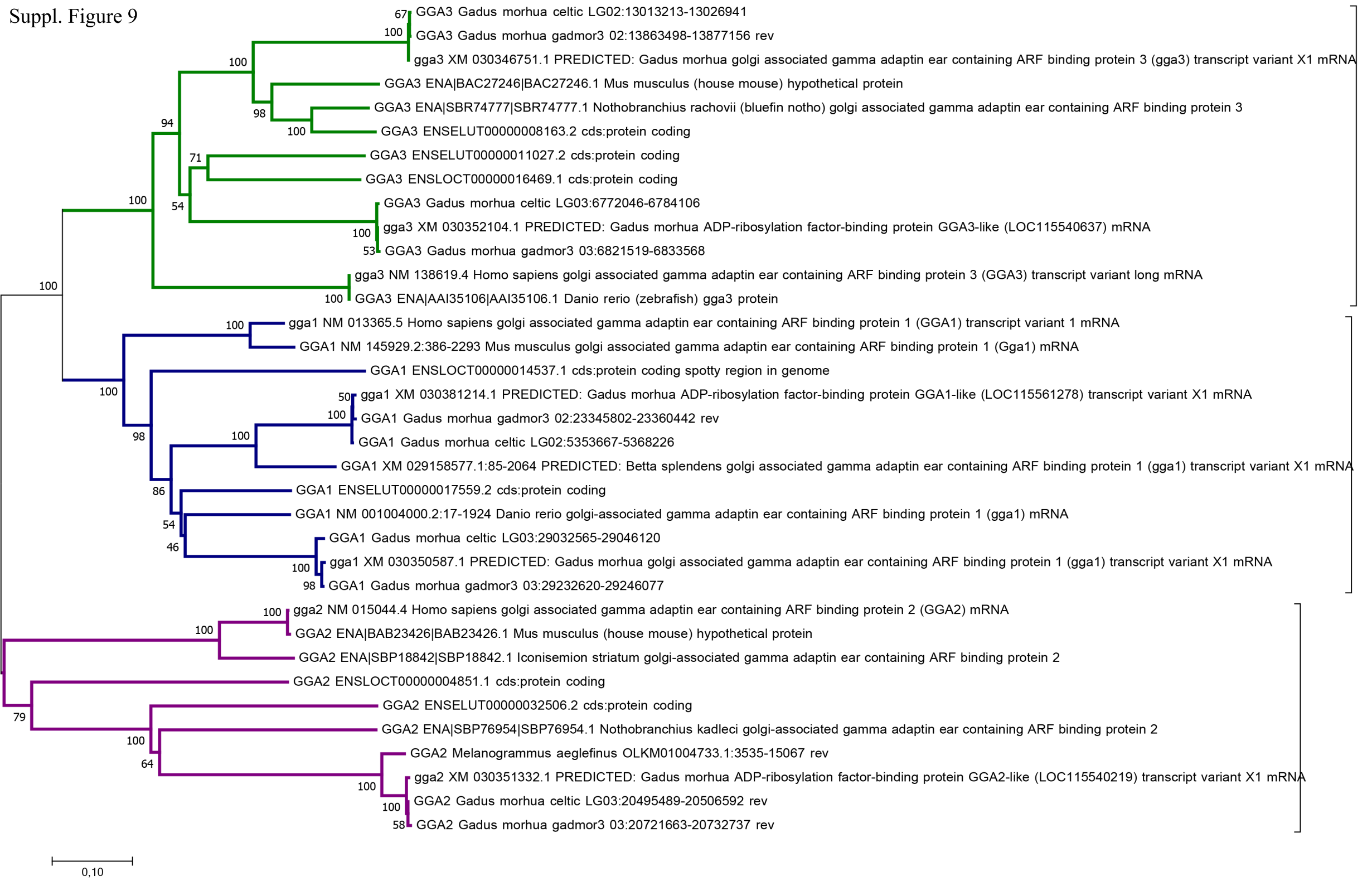

Supplement: Supplementary file 5 [file Image9.pdf]

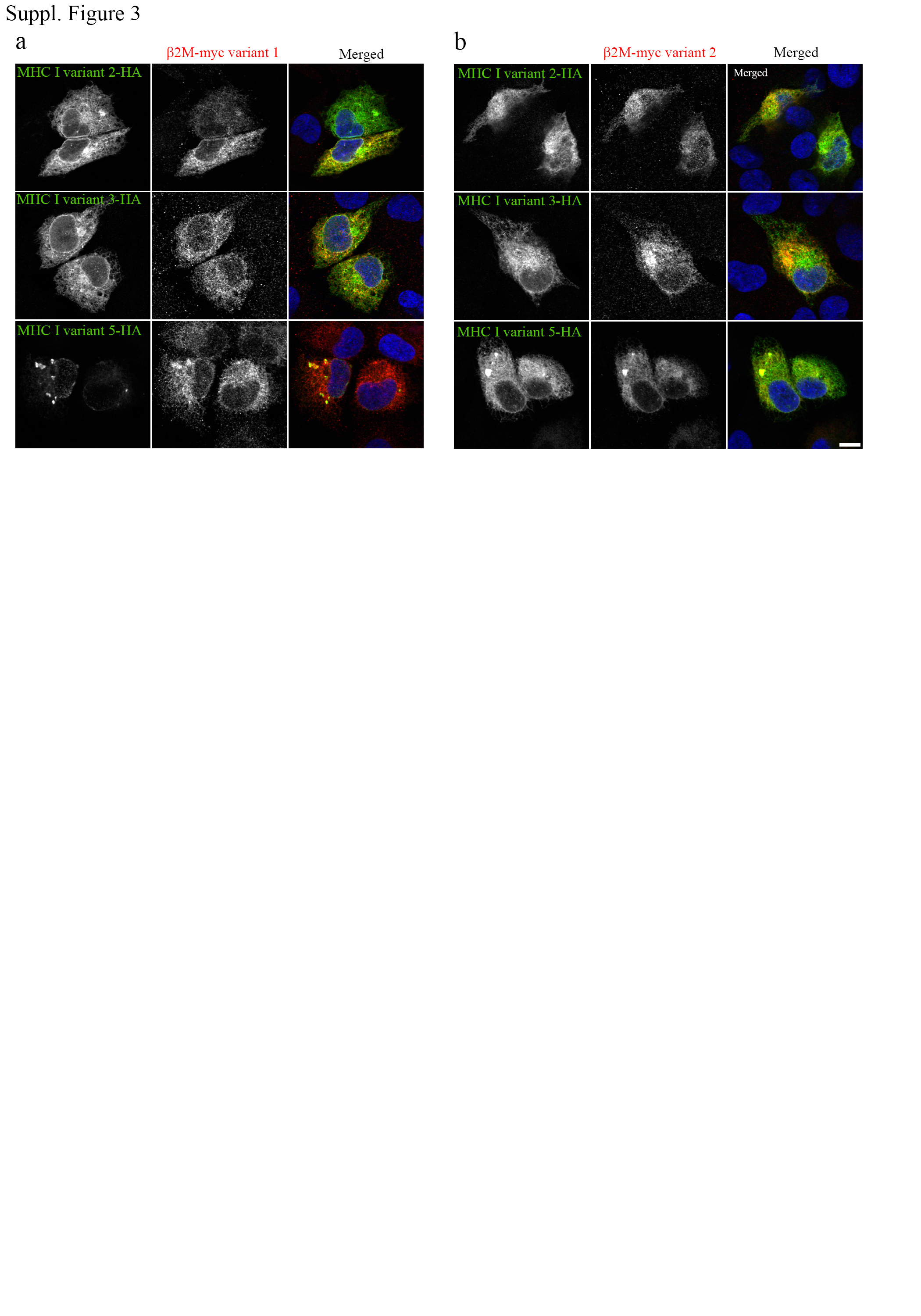

Supplement: Supplementary file 6 [file Image3.tif]

Suppl. Figure 10

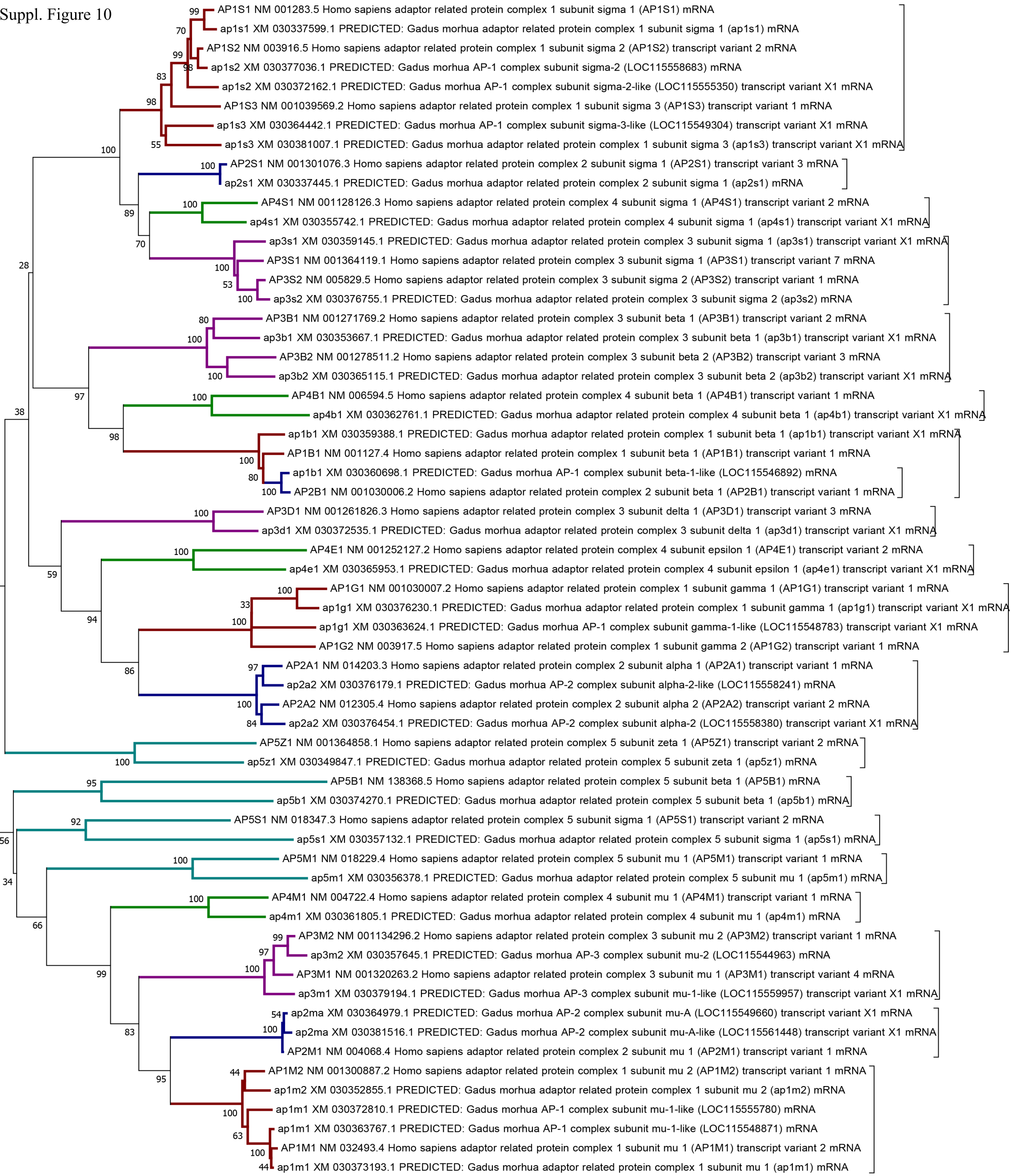

0,20

Supplement: Supplementary file 7 [file Image10.pdf]

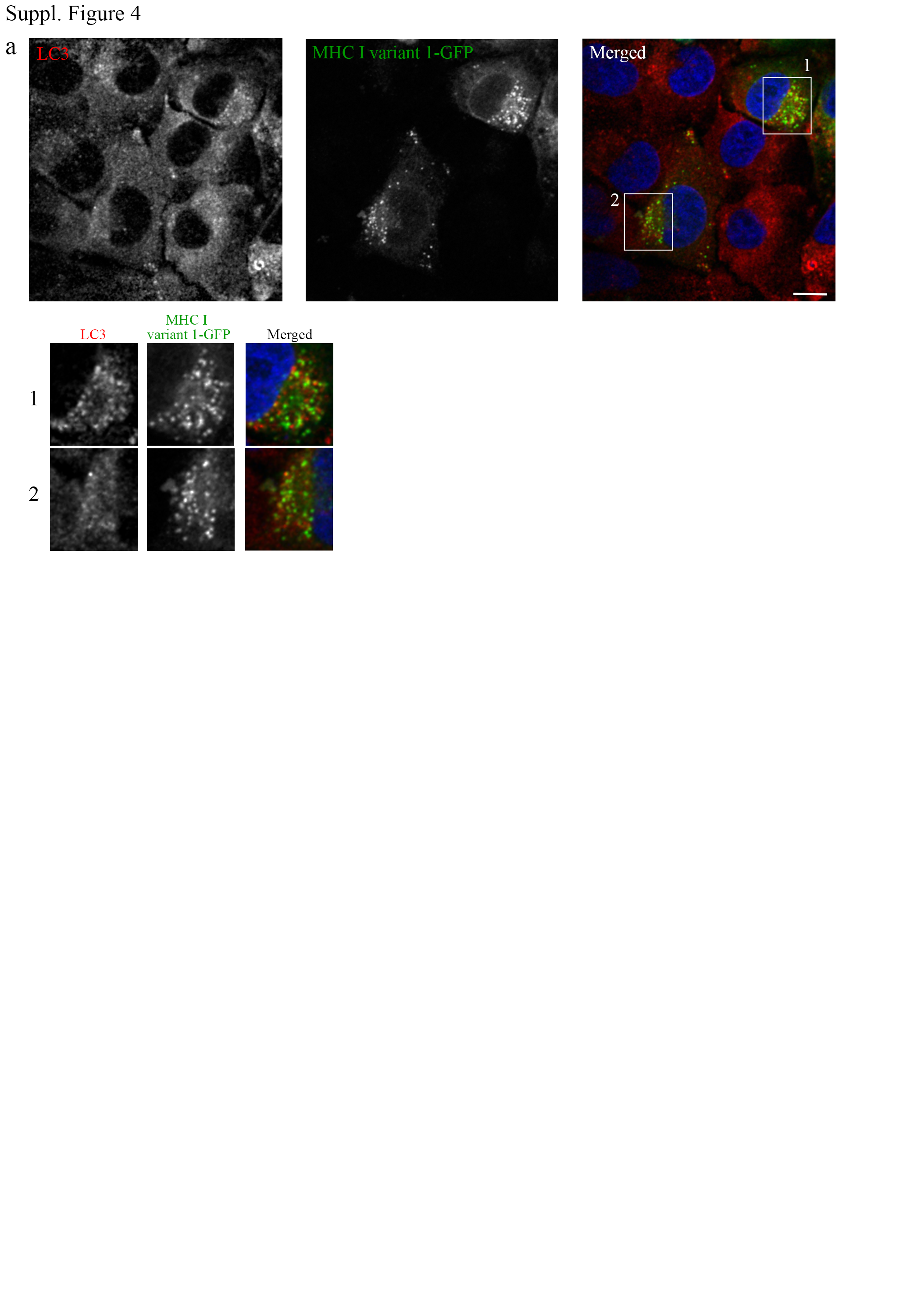

Supplement: Supplementary file 8 [file Image4.tif]

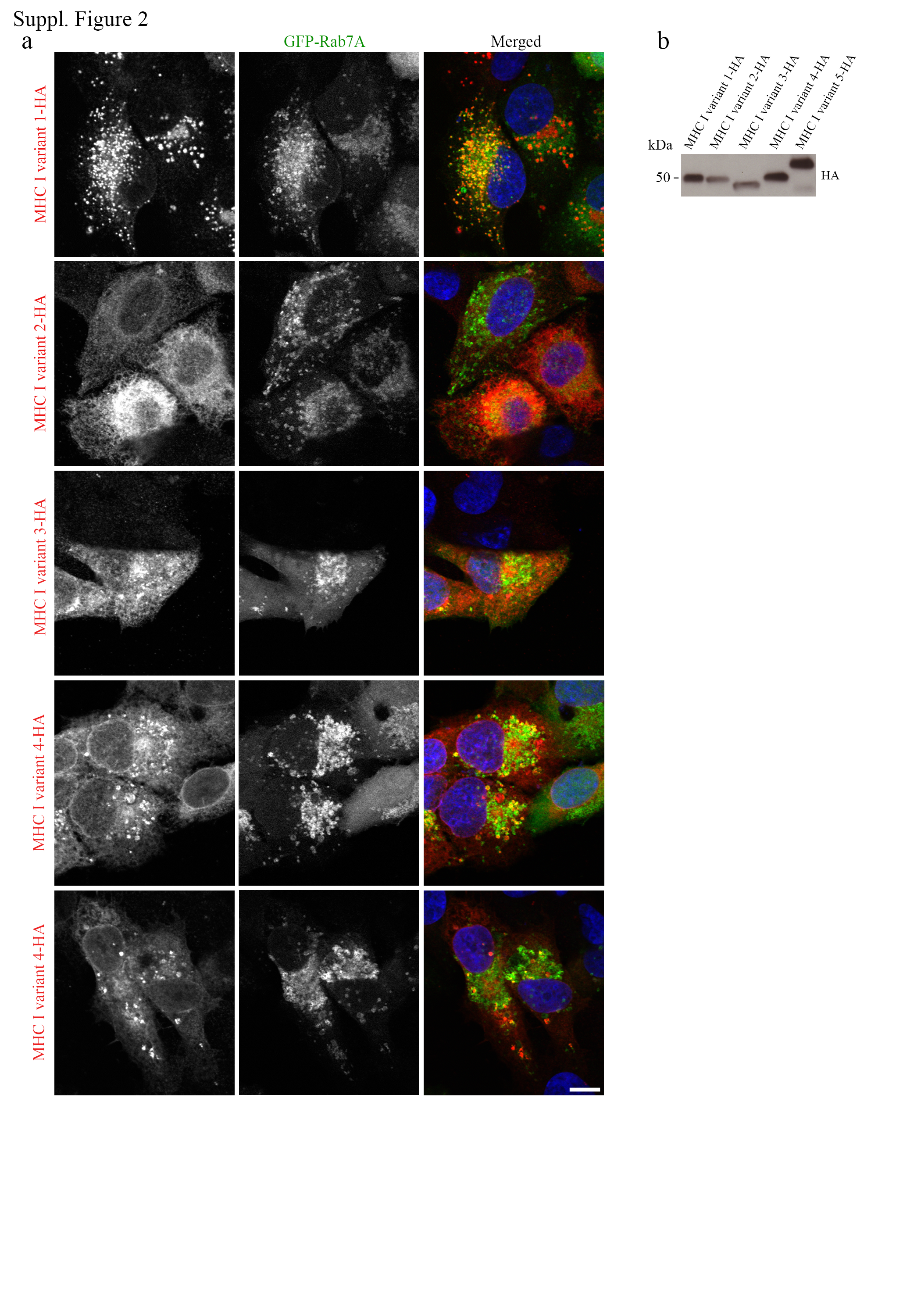

Supplement: Supplementary file 9 [file Image2.tif]

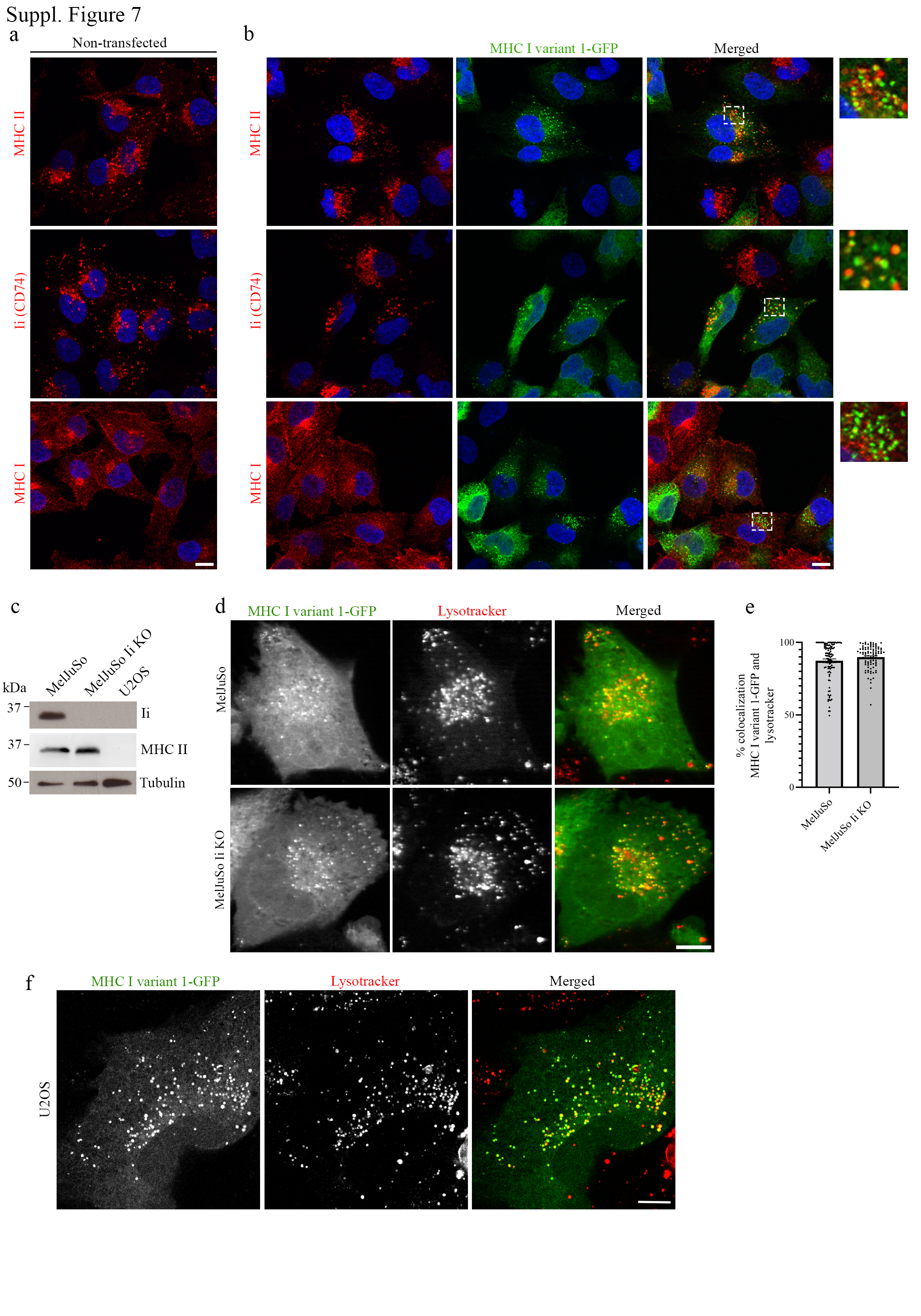

Supplement: Supplementary file 11 [file Image7.tif]

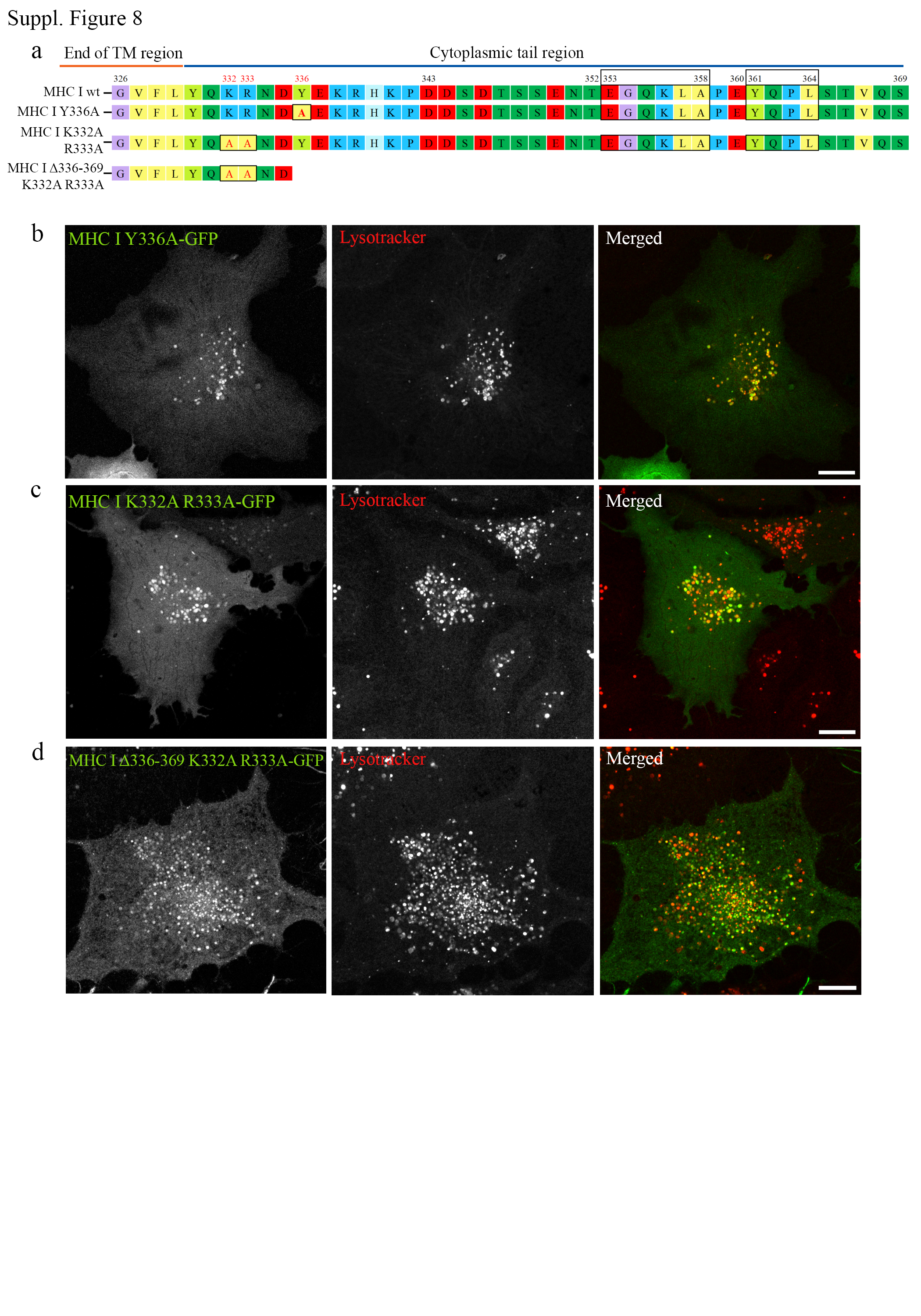

Supplement: Supplementary file 15 [file Image8.tif]

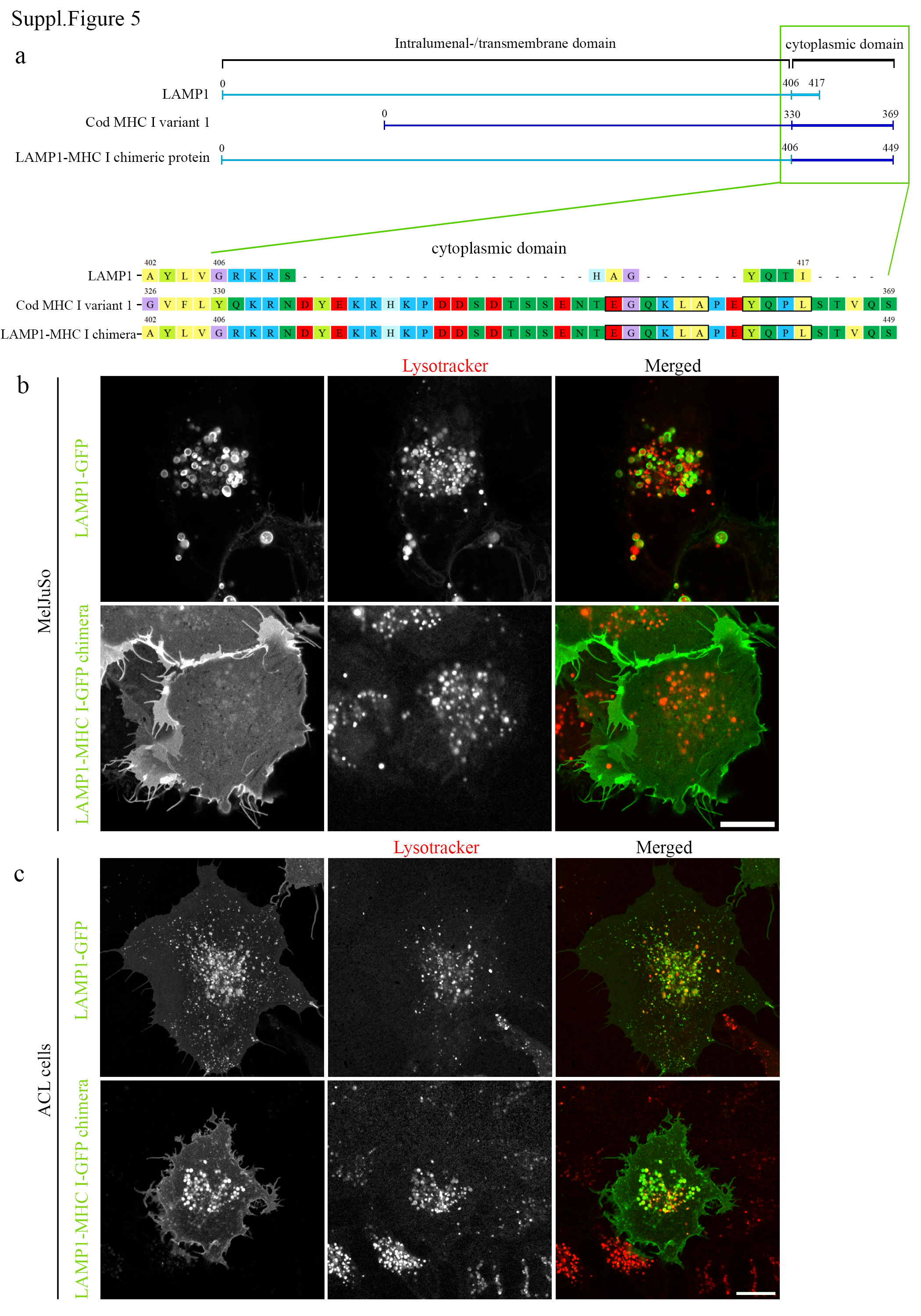

Supplement: Supplementary file 16 [file Image5.tif]

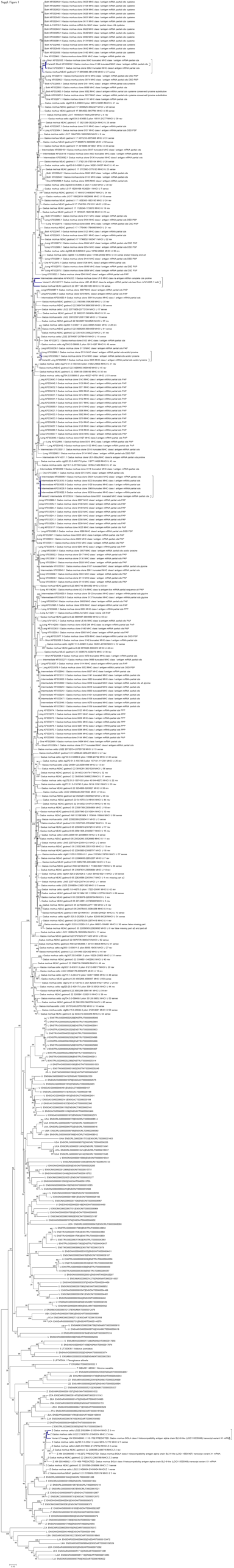

Supplement: Supplementary file 19 [file Image1.pdf]
