# Supplementary material for: Atlantic cod (Gadus morhua) MHC I localizes to endolysosomal compartments independently of cytosolic sorting signals
Source: Front Cell Dev Biol. 2023 Jan 25;11:1050323. doi: 10.3389/fcell.2023.1050323 (PMC9905690; doi:10.3389/fcell.2023.1050323)
Supplement: Supplementary file 13 [file Table3.DOCX]

| Mutant generated | Template | Primer 1 (5’-3’) | Primer 2 (5’-3’) |
| --- | --- | --- | --- |
| MHC I Y361A | MHC I variant 1 | gaagctggctcctgaggcccaacctctgtccaca | tgtggacagaggttgggcctcaggagccagcttc |
| MHC I L357A | MHC I variant 1 | ggtactcaggagccgccttctgcccctcag | ctgaggggcagaaggcggctcctgagtacc |
| MHC I L357A Y361A | MHC I Y361A | ggcctcaggagccgccttctgcccctca | tgaggggcagaaggcggctcctgaggcc |
| MHC I Y336A | MHC I variant 1 | gtttgtgacgcttttcggcatcgttcctcttctggtacagaa | ttctgtaccagaagaggaacgatgccgaaaagcgtcacaaac |
| MHC I Y336A L357A Y361A | MHC I L357A Y361A | gtttgtgacgcttttcggcatcgttcctcttctggtacagaa | ttctgtaccagaagaggaacgatgccgaaaagcgtcacaaac |
| MHC I Δ360-369 | MHC I variant 1 | gccggtggagccaggagccagcttctg | cagaagctggctcctggctccaccggc |
| MHC I Δ352-369 | MHC I Δ360-369 | ccagctctgagaacggctccaccggctc | gagccggtggagccgttctcagagctgg |
| MHC I Δ343-369 | MHC I Δ352-369 | cgaaaagcgtcacaaaccaggctccaccgg | ccggtggagcctggtttgtgacgcttttcg |
| MHC I Δ336-369 | MHC I Δ343-369 | ccggtggagccatcgttcctcttctggtacag | ctgtaccagaagaggaacgatggctccaccgg |
| MHC I Δ332-369 | MHC I Δ336-369 | gtctttctgtaccagggctccaccggctcc | ggagccggtggagccctggtacagaaagac |
| MHC I Δ336-369 K332A R333A | MHC I Δ336-369 | ccggtggagccatcgttcgccgcctggtacagaaagactcc | ggagtctttctgtaccaggcggcgaacgatggctccaccgg |
| MHC I K332A R333A | MHC I variant 1 | ttgttggagtctttctgtaccaggcggcgaacgattacgaaaagcgtcaca | tgtgacgcttttcgtaatcgttcgccgcctggtacagaaagactccaacaa |

**Suppl. Table 3:** Overview of DNA templates and primers used to generate the different point- and deletion mutants of Atlantic cod MHC I variant 1.
