# Supplementary material for: Atlantic cod (Gadus morhua) MHC I localizes to endolysosomal compartments independently of cytosolic sorting signals
Source: Front Cell Dev Biol. 2023 Jan 25;11:1050323. doi: 10.3389/fcell.2023.1050323 (PMC9905690; doi:10.3389/fcell.2023.1050323)
Supplement: Supplementary file 18 [file DataSheet1.docx]

**Supplementary Material**

**Supplementary Figure 1. Inferred unrooted phylogenetic tree of all MHC class I U lineage cDNA clones and predicted Z lineage transcripts obtained from NCBI.**

Reference sequences added from REF Grimholt representing teleost species, and Gadmor3/Celtic genome. Tree was generated using the neighbour joining method, Poisson distribution and 500 bootstrap replicates (bootstrap values are shown at the nodes). Generated constructs are annotated as variant 1-5, and their respective subclades are colored in dark blue. Tree resolves according to the major MHC lineages known in teleost fish (U, L, S, P and Z), Atlantic cod only has U and Z lineage genes.

**Supplementary Figure 2. Intracellular localization of five Atlantic cod MHC I HA-tagged variants in MelJuSo cells.**

(a) Representative images of MelJuSo cells transiently co-transfected with Atlantic cod MHC I variants tagged with HA (red), and the endolysosomal marker GFP-Rab7A (green). Colocalization between green and red channels results in yellow signal in the merged panels Cells were imaged using an Olympus FluoView 1000 microscope. Scale bar: 10 μm. (b) MelJuSo cells were transiently transfected with HA-tagged Atlantic cod MHC I variants, lysed and subjected to Western blot analysis using antibodies against HA.

**Supplementary Figure 3. Atlantic cod MHC I variant 2, 3 and 5 localization in MelJuSo cells is not affected by the expression of Atlantic cod β‑2 microglobulin.**

(a) Representative images of MelJuSo cells transiently co-transfected with Atlantic cod β‑2 microglobulin (β2M) variant 1-Myc and either Atlantic cod MHC I variant 2-HA, Atlantic cod MHC I variant 3‑HA or Atlantic cod MHC I variant 5‑HA. Cells fixed and stained against HA (green) and Myc (red). Cells were imaged using an Olympus FluoView 1000 microscope. Scale bar: 10 μm. (b) Representative images of MelJuSo cells transiently co-transfected with Atlantic cod β‑2 microglobulin (β2M) variant 2-Myc and either Atlantic cod MHC I variant 2-HA, Atlantic cod MHC I variant 3‑HA or Atlantic cod MHC I variant 5‑HA. Cells fixed and stained against HA (green) and Myc (red). Cells were imaged using an Olympus FluoView 1000 microscope. Scale bar: 10 μm.

**Supplementary Figure 4. Atlantic cod MHC I does not localize to autolysosomes in MelJuSo cells.**

(a) Representative images of MelJuSo cells transiently transfected with Atlantic cod MHC I variant 1-GFP (green), fixed and stained for the autolysosomal marker LC3 (red). Cells were imaged using an Olympus FluoView 1000 microscope. Scale bar: 10 μm. Magnification of boxed areas shown below.

**Supplementary Figure 5. Chimeric LAMP1-MHC I variant 1 re-localizes to the plasma membrane in MelJuSo cells but partly retains its endolysosomal localization in ACL cells.**

(a) Schematic representation of the chimeric LAMP1 and MHC I variant 1 construct consisting of the intraluminal and transmembrane domains of human LAMP1 together with the cytosolic tail of the Atlantic cod MHC I variant 1. Amino acid sequences of cytoplasmic domains are shown with putative dileucine and tyrosine sorting signals boxed. Numbers above the sequences mark amino acid positions of the proteins. (b) Representative images of MelJuSo cells transiently transfected with LAMP1-GFP or LAMP-MHC I-GFP chimera (green). The cells were stained with lysotracker red and imaged using the Zeiss LSM880 Fast AiryScan microscope. Colocalization between green and red channels results in yellow signal in the merged panels. Scale bar: 10 μm. (c) Representative images of ACL cells transiently transfected with LAMP1-GFP or LAMP-MHCI-GFP chimera (green). The cells were stained with lysotracker red and imaged using the Zeiss LSM880 Fast AiryScan microscope. Scale bar: 10 μm.

**Supplementary Figure 6. Chimeric Atlantic cod-human MHC I variant 1 retains its endolysosomal localization in both MelJuSo and ACL cells.**

(a) Schematic representation of Atlantic cod-human MHC I variant 1 chimeric construct consisting of the intraluminal and transmembrane domains of Atlantic cod MHC I variant 1 together with the cytosolic tail of human MHC I (HLA-A2). Amino acid sequences of cytoplasmic domains are shown with putative dileucine and tyrosine sorting signals boxed. Numbers above the sequences mark amino acid positions of the proteins. (b) Representative images of MelJuSo cells transiently transfected with HLA-A2-GFP (green) or chimeric Atlantic cod-human MHC I variant 1-GFP (green). The cells were stained with lysotracker red and imaged using a Zeiss LSM880 Fast AiryScan microscope. Colocalization between green and red channels results in yellow signal in the merged panels. Scale bar: 10 μm. (c) Representative images of ACL cells transiently transfected with HLA-A2-GFP (green) or chimeric Atlantic cod-human MHC I variant 1-GFP (green). The cells were stained with lysotracker red and imaged using a Zeiss LSM880 Fast AiryScan microscope. Colocalization between green and red channels results in yellow signal in the merged panels. Scale bar: 10 μm.

**Supplementary Figure 7. Atlantic cod MHC I localizes to endolysosomes in MelJuSo cells independently of MHC class II trafficking machinery.**

(a) Representative images of MelJuSo cells fixed and immunostained for MHC II, Ii (CD74) or MHC I. Cells were imaged using an Olympus FluoView 1000 microscope. Scale bar: 10 μm. (b) Representative images of MelJuSo cells transiently transfected with Atlantic cod MHC I variant 1-GFP (green), fixed and immunostained for MHC II, Ii (CD74) or MHC I (red). Cells were imaged using an Olympus FluoView 1000 microscope. Scale bar: 10 μm. Magnifications of the boxed areas are shown in the insets. (c) Western blot analysis of lysates from MelJuSo, MelJuSo Ii knock-out (KO) and U2OS cells using the indicated antibodies. (d) Representative images of MelJuSo and MelJuSo Ii KO cells transiently transfected with human Atlantic cod MHC I variant 1-GFP (green). Cells were stained with lysotracker red and imaged using an Andor Revolution XD Spinning Disk microscope. Colocalization between green and red channels results in yellow signal in the merged panels. Scale bar: 10 μm. (e) The graph represents the percentage of Atlantic cod MHC I variant 1-GFP vesicles positive for lysotracker red. Colocalization was done by object-based analysis using ImageJ software. Values represent the mean ± s.e.m. from at least three independent experiments. n ≥ 94 cells. Non-significant (Two-tailed paired Student´s t-test). (f) Representative images of U2OS cells transiently transfected with Atlantic cod MHC I variant 1-GFP (green). The cells were stained with lysotracker red and imaged using Zeiss LSM880 Fast AiryScan microscope. Colocalization between green and red channels results in yellow signal in the merged panels. Scale bar: 10 μm.

**Supplementary Figure 8. Atlantic cod MHC I Y336A,MHC I K332A R333A and MHC I K332A R333A Δ336-369 localize to acidic compartments in ACL cells.**

(a) Amino acid sequences of Atlantic cod MHC I variant 1 wild type (wt), MHC I Y336A, MHC I K332A R333A and MHC I Δ336-369 K332A R333A Δ336-369. All substitutions are marked with black box and red letters. The numbers above the sequences mark the amino acid positions. (b) Representative image of an ACL cell transiently transfected with Atlantic cod MHC I Y336A-GFP (green), stained with lysotracker red and imaged using a Zeiss LSM880 Fast AiryScan microscope. Colocalization between green and red channels results in yellow signal in the merged panels. Scale bar: 10 μm. (c) Representative image of an ACL cell transiently transfected with MHC I K332A R333A-GFP (green). The cell was stained with lysotracker red and imaged using a Zeiss LSM880 Fast AiryScan microscope. Scale bar: 10 μm. (d) Representative image of an ACL cell transiently transfected with MHC I Δ336-369 K332A R333A Δ336-369-GFP (green), stained with lysotracker red and imaged using a Zeiss LSM880 Fast AiryScan microscope. Scale bar: 10 μm.

**Supplementary Figure 9. Inferred unrooted phylogenetic tree of all Atlantic cod GGAs.**

Unrooted evolutionary tree inferred from the protein alignment of selected GGAs using the Neighbor joining method, Poisson distribution and 500 bootstrap replicates. Atlantic cod sequences cluster together with representatives from other species indicating common ancestry. For the corresponding fasta file, see Supplementary Data sheet 5.

**Supplementary Figure 10. Inferred unrooted phylogenetic tree of all Atlantic cod APs.**

Unrooted evolutionary tree inferred from the protein alignment of selected APs using the Neighbor joining method, Poisson distribution and 500 bootstrap replicates. Atlantic cod sequences cluster together with representatives from other species indicating common ancestry. For the corresponding fasta file, see Supplementary Data sheet 6.

**Supplementary Table 1. Alignment analysis between human and Atlantic cod adaptor proteins.**

Table overviewing the investigated adaptor protein families and their representatives in Atlantic cod and humans. Candidate gene names with descriptions are presented from the annotation of the latest Atlantic cod genome assembly together with the annotated gene models from the human genome reference. The 5th column contains a description of the overall alignment between Atlantic cod and human sequences complemented by the last column presenting a subset of calculated p-distance values (if multiple human gene copies, p-distances towards only one copy is presented). The p-distance values are derived from pair-wise calculations on protein sequences indicating the proportion of dissimilar amino acids between two sequences. For all calculated p-distances see Supplementary Table 2.

**Supplementary Table 2. P-distances**

Overview of all p-distances calculated for pair-wise contrasts of all human and Atlantic cod GGAs / APs protein sequences. For the corresponding fasta files see Supplementary Data sheet 3 (for GGAs) and Supplementary Data sheet 4 (for APs).

**Supplementary Table 3. DNA templates and primers used to generate point- and deletion mutations.**

Table indicating which primers were used to create the different point- and deletion mutations in Atlantic cod MHC I variant 1.

**Supplementary Table 4.**

Table including GenBank accession numbers.

S**upplementary. Data sheet 3. Fasta file for p-distance calculation of GGAs.**

Multiple protein sequence alignment of human and cod GGAs sequences aligned using muscle.

S**upplementary. Data sheet 4. Fasta file for p-distance calculation of APs.**

Multiple protein sequence alignment of human and cod APs sequences aligned using Muscle.

**Supplementary Data sheet 5. Fasta file for phylogenetic tree of Atlantic cod GGAs**

Multiple protein sequence alignment of human and cod GGAs together with selected protein sequences of GGAs from other species aligned using Muscle.

**Supplementary Data sheet 6. Fasta file for phylogenetic tree of Atlantic cod APs**

Multiple protein sequence alignment of human and cod APs together with selected protein sequences of APs from other species aligned using Muscle.
